# The role of immune checkpoint molecules in PRRSV-2-induced immune modulation: insights from comparative *in vivo* evaluation including NADC34-like PRRSV

**DOI:** 10.1128/jvi.02298-24

**Published:** 2025-06-03

**Authors:** Seung-Chai Kim, Hwan-Ju Kim, Sang Chul Kang, Aarif Rasool, Ji-Hyun Ryu, Jung-Min Lee, Won-Il Kim

**Affiliations:** 1 College of Veterinary Medicine, Jeonbuk National University 65432, Iksan, Republic of Korea; 2 Optipharm Co Ltd. 676477 , Cheongju, Republic of Korea; University of Michigan Medical School, Ann Arbor, Michigan, USA

**Keywords:** porcine reproductive and respiratory syndrome virus, PRRSV, NADC34, pathogenicity, immunopathogenesis, immunosuppression, immune checkpoint molecules, IDO1, kynurenine pathway

## Abstract

The emergence of genetically diverse PRRSV-2 lineages, including the NADC34-like virus, presents significant challenges to the swine industry due to their variable pathogenicity and impact on immune modulation. However, the precise mechanisms underlying virus-induced immune modulation remain poorly understood. This study investigated the immunopathological characteristics of the Korean NADC34-like PRRSV strain JBNU-22-N01 in comparison with the NADC30-like strain PJ73 and the prototype strain VR2332 in a weaned piglet model. All PRRSV-2 strains exhibited moderate pathogenicity without mortality, following a similar infection course characterized by interstitial pneumonia, cytokine cascades (e.g., IFN-α, IFN-γ, IL-1β, IL-12p40, IL-10, CCL2, CCL5, CCL8, and CXCL10), and upregulation of interferon-stimulated genes [e.g., ISG12(A)]. Flow cytometry analysis revealed significant alterations in the composition of bronchoalveolar lavage (BAL) cell populations, including a decreased frequency of alveolar macrophages and an increased proportion of infiltrating immune cells (monocyte-derived cells, T cells, and NK cells). However, the majority of infiltrated CD4^+^ and CD8^+^ T cells exhibited minimal expression of effector cytokines (e.g., IFN-γ), suggesting a phenotypically naïve-like or functionally unresponsive state. The mRNA expression levels of BAL cells revealed significant upregulation of immune checkpoint molecules (e.g., PD1, PDL1, CTLA4, LAG3, and IDO1), indicating immune regulation potentially mediated through cell-to-cell communication. Notably, IDO1 expression was modestly associated with increased systemic Kyn/Trp ratios, supporting the hypothesis that the kynurenine pathway may contribute to systemic immune modulation during PRRSV infection. From a strain-specific perspective, JBNU-22-N01 displayed a faster replication rate, resulting in an accelerated and heightened infection process with stronger immune responses. It uniquely induced robust expression of immune checkpoint molecules and enhanced IDO1 protein production in bronchoalveolar lavage cells, highlighting the immunomodulatory potential of NADC34-like PRRSV. These findings offer insights into both general and strain-specific immunopathogenesis of PRRSV-2 and support the development of better treatments.

**IMPORTANCE:**

Porcine reproductive and respiratory syndrome virus (PRRSV) is a major pathogen affecting the swine industry; however, its immune-related mechanisms remain incompletely understood. Here, we analyzed how three genetically distinct PRRSV strains, including the globally prevalent NADC34-like strain, interact with the immune system in piglets. Our results showed that PRRSV induces severe lung inflammation accompanied by immune cell infiltration. However, many infiltrating immune cells remained inactive, likely due to increased expression of immune-suppressive molecules. Among these, the enzyme indoleamine 2,3-dioxygenase-1 (IDO1) was notably upregulated, activating a metabolic pathway linked to immune regulation and suppression. The NADC34-like strain displayed a faster replication rate, leading to more rapid immune responses and stronger suppression compared with the other strains. These findings provide new insights into how PRRSV manipulates the immune system and suggest potential targets for improved prevention and treatment strategies.

## INTRODUCTION

Porcine reproductive and respiratory syndrome (PRRS) has become a critical global viral epidemic, causing substantial economic losses since it was first identified in the United States in 1987 (1, 2). The disease’s causative agent, PRRS virus (PRRSV), is an enveloped, single-stranded positive-sense RNA virus belonging to the order *Nidovirales*, family *Arteriviridae*, with a genome approximately 15 kb in length. PRRSV comprises two genotypes: PRRSV-1 (European type, prototype strain Lelystad virus) and PRRSV-2 (North American type, prototype strain VR2332), recently designated as *Betaarterivirus suid 1* and *Betaarterivirus suid 2*, respectively (ICTV2021).

RNA viruses, including PRRSV, exhibit high mutation rates due to the lack of 3’ to 5’ exonuclease proofreading in RNA-dependent RNA polymerase (RdRp). Among reported RNA viruses, PRRSV has one of the highest calculated nucleotide substitution rates (3, 4). PRRSV encodes at least 11 open reading frames (ORFs), with the ORF5 gene coding for the major envelope glycoprotein GP5. This protein is essential for viral assembly, infectivity, and the induction of virus-neutralizing antibodies and exhibits significant genetic diversity across strains (5–7). Based on ORF5 gene phylogeny, PRRSV-2 strains have been systematically classified into 11 genetic lineages (L1-L11) and 21 sublineages (L1A-L1F, L1H-L1J, L5A-L5B, L8A-L8E, and L9A-L9E) (8).

Distinct lineages of PRRSV-2 demonstrate varying levels of pathogenicity and immune responses, likely due to their genetic and antigenic diversity. Highly virulent PRRSV strains, such as the Chinese highly pathogenic PRRSV (HP-PRRSV; L8E), result in elevated viremia and tissue viral loads, causing high mortality and severe clinical manifestations (9, 10). Recently, virulent sublineages of L1 PRRSV-2, particularly L1C (NADC30-like) and L1A (NADC34-like), have expanded globally, including in the U.S. and China (9, 11). The NADC30 strain, which exhibits a distinct “111 + 1 + 19” amino acid deletion pattern in the nonstructural protein 2 (NSP2) region, was identified in the U.S. in 2008 from the outbreak of severe respiratory distress in piglets (12, 13). The RFLP 1-7-4 strains or NADC34-like strains, which emerged in the U.S. in 2014, were characterized by high mortality among newborn piglets and abortion “storms” in sow herds (14, 15). These NADC34-like strains, with the distinct 100-aa deletion pattern in NSP2, have been subsequently reported from outbreaks in other regions, including Korea in 2022 (16), posing the risk of a novel epidemic lineage 1 PRRSV-2.

PRRSV primarily targets porcine alveolar macrophages (PAMs) in the lungs through the CD163 receptor, affecting both circulating monocytes and monocyte-derived cells to a lesser extent (17, 18). Immunohistochemistry staining of lung sections (19) and flow cytometry of bronchoalveolar lavage (BAL) cells (20, 21) have shown that no more than 2% of PAMs harbor viral antigens, although it is hypothesized that PRRSV induces the death of both directly infected cells and uninfected bystander cells (22, 23). In infected piglets, viremia typically peaks at around 7–10 days post-infection (dpi) and resolves by approximately 28 dpi, although this could vary by strain (24, 25). PRRSV infection triggers dysregulated immune responses, characterized by delayed peripheral T cell activation that becomes evident after 3 weeks post-infection (26, 27). Adaptive immune responses are similarly delayed, with non-neutralizing antibodies appearing within the first week post-infection, whereas neutralizing antibodies are not typically detected until after 4 weeks (25). Compared with other swine viruses, such as African swine fever virus (ASFV) and swine influenza virus (SIV), PRRSV induces limited systemic inflammatory responses, instead of promoting localized pro-inflammatory cytokine expression linked to interstitial pneumonia development (26–28). Additionally, PRRSV has been associated with immunosuppressive mechanisms involving upregulated regulatory T cells (Tregs) and IL-10 production, although reports on IL-10 expression have been inconsistent (25, 27), leaving critical aspects of PRRSV-induced immunosuppression mechanisms unresolved.

In addition to Tregs and IL-10, recent research has suggested that coinhibitory molecules or “immune checkpoints” may play a role in PRRSV-induced immunosuppression. These molecules, which include PD1, CTLA4, TIM3, and LAG3 on T cells and PDL1 and IDO1 on macrophages and dendritic cells, not only regulate immune responses to prevent excessive tissue damage but may also contribute to immune evasion by pathogens (29–31). Upregulation of these immune checkpoints has been observed in PRRSV-1-infected thymus (32), lung (33), bronchoalveolar lavage (BAL) cell (28), PRRSV-2-infected inguinal lymph node (34), BAL cell (23), and PAMs (20). Despite these preliminary results, mostly derived from transcriptomic analysis, comprehensive studies on these mechanisms in PRRSV-2 infections are limited.

This study highlights the pathogenic and immunological dynamics of PRRSV infections, specifically focusing on the Korean NADC34-like PRRSV strain JBNU-22-N01, and its comparison with Korean NADC30-like isolate PJ73 (JB15-N-PJ73-GN) and PRRSV-2 prototype strain VR2332, across multiple dimensions including clinical presentation, viral load, lung pathology, immune cell dynamics, cytokine and chemokine responses, and the activation of immune checkpoint molecules. The phenotypic difference of NADC34-like PRRSV was analyzed, and general immunosuppressive mechanisms induced by PRRSV-2 were investigated, specifically by examining immune responses in the lung during the early stages of infection.

## MATERIALS AND METHODS

### Virus and cells

The Korean PRRSV-2 strains JBNU-22-N01 (GenBank: OP970983), the first NADC34-like PRRSV (L1A) isolated in Korea (16), and PJ73 (GenBank: MZ287317), Korean NADC30-like PRRSV (L1C) strain, as well as PRRSV-2 prototype strain VR2332 (GenBank: AY150564), were selected for infection experiments based on their genetic characteristics (12, 16) and pathogenicity (35). Viral propagation was conducted in primary PAM cells derived from PRRSV-negative, 4-week-old piglets maintained in our lab, as previously described (16). In brief, cells were cultured in Gibco RPMI-1640 medium (Life Technologies, USA) with 10% heat-inactivated FBS, 2 mM l-glutamine, and 1× Anti-Anti solution (100 IU/mL penicillin, 100 µg/mL streptomycin, 0.25 µg/mL amphotericin B; Life Technologies) in a 5% CO2 chamber at 37°C. Viral titers were determined by cytopathic effect (CPE) and expressed as TCID_50_/mL, following established protocols (24).

### Animal study

Twenty-four 4-week-old weaned piglets, purchased from a PRRSV-seronegative farm, were randomly assigned to four groups (*n* = 6 per group). Each group was assigned for inoculation with VR2332, PJ73, JBNU-22-N01, or PBS (negative control). Each group was housed separately. After a 3-day acclimatization period, pigs were inoculated intramuscularly (IM) with 2 mL of virus inoculum (1 × 10^3^ TCID₅₀/mL in sterile PBS) or, for the negative control group, with sterile PBS alone. The IM route was selected to ensure consistent delivery of a defined viral dose to each pig, minimizing variability introduced by mucosal clearance or respiratory deposition that can occur with intranasal (IN) inoculation. IM inoculation has been widely used in PRRSV experimental models to assess immune responses and pathogenesis in a controlled setting (20, 23, 24).

Three pigs from each group were euthanized humanely on days 7 and 14 post-inoculation (dpi). Euthanasia was performed by electrocution following intramuscular injection of 1 mL of azaperone (40 mg/mL, StressGuard, Dong Band Inc., Republic of Korea). Lung, bronchial lymph node (BLN), and tonsil samples were aseptically collected. The left lung lobe was clamped and sampled for RNA extraction and cytokine assays, as well as for histopathological analysis by preserving in 10% neutral-buffered formalin (NBF). Blood samples and nasal swabs were collected at 0, 4, 7, and 14 dpi, and body weights were recorded at each time point. Body temperature was measured daily using a subcutaneously implanted microchip (Biotherm 13 Pit Tag, Biomark, USA) and a microchip reader (Destron Fearing, USA).

### Quantification of viral load

Serum viremia, nasal viral shedding, and viral load in lung, BLN, and tonsil tissues were measured at 0, 4, 7, and 14 dpi. For tissue samples, 0.1 g of tissue was minced, diluted in PBS (1:10), and homogenized using a Beadbeater TissueLyser II (QIAGEN). Viral RNA was extracted from 200 µL of serum or tissue homogenate supernatants using a Tianlong automatic nucleic acid extractor (NP968, Xi’an Tianlong Science & Technology Co., China). RT-qPCR was performed with a Prime-Q PCV2/PRRSV Detection Kit (Genet Bio, Republic of Korea) on a CFX96 Real-Time PCR Detection System (Bio-Rad, USA) to quantify serum viremia and tissue viral loads. Virus titers (TCID₅₀/mL) were calculated from Cq values by generating a standard curve for each virus.

### Anti-PRRSV-specific IgG ELISA

Serum samples were tested for anti-PRRSV IgG antibodies using a commercial ELISA kit (PRRS Ab ELISA 4.0; BioNote Inc., Republic of Korea) following the manufacturer’s instructions. Samples with an S/P ratio ≥0.4 were considered positive for PRRSV antibodies.

### Pathological evaluation of the lung

Lung lobes fixed in NBF were embedded in paraffin and sectioned (6 µm). Sections were stained with hematoxylin and eosin (HE) and examined microscopically for histopathological scoring, following a standardized scoring system (36) (Table S1). PRRSV immunohistochemical (IHC) staining was conducted based on a previous study (37), with modification of using anti-PRRSV monoclonal antibody 4A5 (MEDIAN Diagnostics Inc., Republic of Korea). Antigen amount was given a ranked score of 0–4 as an estimate of the number of positive cells per lung section from each block and termed as lung IHC score: 0 = no PRRSV-antigen-positive cells, 1 = 1–10 positive cells, 2 = 11–30 positive cells, 3 = 31–100 positive cells, and 4 = >100 positive cells.

### Isolation of BAL cells and PBMC

After collecting tissue samples from clamped left lung lobes, the right lung was lavaged with 75 mL of sterile PBS supplemented with Anti-Anti and 100 µg/mL ampicillin (ThermoFisher Scientific, USA). The collected lavage fluid was centrifuged at 1,000 × *g* at room temperature for 10 min to separate BAL cells for flow cytometry and quantification of mRNA expressions. The BAL cells were washed three times with the same PBS solution used for lavage and snap-frozen using liquid nitrogen and stored immediately at −80°C for further usage.

PBMCs were isolated by density gradient centrifugation using Leucosep tubes and Leucoprep media (Greiner Bio-One North America Inc., USA) following the manufacturer’s protocols. Blood was stratified on Leucoprep solution at a 1:1 ratio and centrifuged at 1,000 × *g* for 10 min. The purified PBMCs were collected, and contaminating red blood cells (RBCs) were removed by treatment with RBC lysis buffer (eBioscience, USA). RBC-removed PBMCs were washed twice with sterile PBS and stored immediately at −80°C for further usage.

### Flow cytometry of BAL cells and PBMC

The collected BAL cells were monitored using flow cytometry for phenotypic changes and infiltration of T cell subsets into the lungs. BALF cells (1 × 10^6^ cells) were transferred to microtiter plates and washed with FACS buffer (3% FBS in PBS and 0.02% sodium azide) for 5 min at 1,500 rpm. The cells were then triple- or quadruple-stained for swine membrane markers and/or internuclear and intranuclear markers (Table S2) as previously described (24, 38, 39). First, BAL cells were stained with Zombie Green live-dead fixable dye staining, followed by porcine MHCII, CD163, and CD172α (Sirpα) surface markers to distinguish live/dead PAMs. From the MHCII^+^ cell population, five phenotypically and functionally defined subpopulations were distinguished using CD163 and CD172α as follows based on previous studies: MHCII^+^/CD172α^+^/CD163^high^ (alveolar macrophages; AMɸ), MHCII^+^/CD172α^+^/CD163^int^ (monocyte-derived macrophages; moMɸ), MHCII^+^/CD172α^+^/CD163^low^ (monocyte-derived dendritic cells; moDC), MHCII^+^/CD172α^+^/CD163^-^ (conventional dendritic cells 2; cDC2), and MHCII^+^/CD172α^-^_/_CD163^-^ (conventional dendritic cells 1; cDC1) (24, 38).

For the T cell population, BAL cells and PBMC were stained with porcine CD3 and T cell receptor (TCR)1δ antibodies to distinguish CD3^+^/TCRγδ^-^ (αβT) cells and CD3^+^/TCRγδ^+^ (γδT) cells. Also stained with porcine CD4α, CD8α antibodies, αβ T were further distinguished into CD3^+^/TCRγδ^-^/CD4^+^/CD8^-^ cell (CD4^+^ αβ T), and CD3^+^/TCRγδ^-^/CD4^-^/CD8^+^ (CD8^+^ αβT) cell. γδT cells were also further distinguished into CD3^+^/TCRγδ^+^/CD8^-^ (CD8^-^ γδT; proinflammatory γδT cells) and CD3^+^/TCRγδ^+^/CD8^+^ (CD8^+^ γδT; regulatory γδT cells), based on a previous study (39). Natural killer (NK) cell subsets and T cell subsets, including regulatory T cells (Tregs), cytotoxic T cells (CTLs), and T-helper cells 1 (Th1) and 17 (Th17), were measured from BAL cells and PBMC as previously described (24). In brief, NKp46^+^ (CD3^-^/CD8^+^/NKp46^+^) and NKp46^-^ NK (CD3^-^/CD8^+^/NKp46^-^) cells were analyzed using only surface staining of porcine CD3, CD8α, and CD335 (NKp46) primary antibodies. For Tregs (CD4^+^/CD8^-^/CD25^+^/FoxP3^+^) and their derivatives, CD4^+^/CD8^-^/CD25^-^/FoxP3^+^ cell, which require intranuclear staining of FoxP3 after cell surface staining, cells were fixed with cold fixation/permeabilization buffer (eBioscience) after cell surface staining of porcine CD4α, CD8α, and CD25. For CTLs (CD3^+^/CD8^+^/IFNγ^+^) and T-helper cell subsets (CD4^+^/CD8^-^/IFNγ^+^; Th1, CD4^+^/CD8^-^/IL17^+^; Th17), which require intracellular staining, single cell suspensions were treated with a mixture of 1 × cell stimulation cocktail (eBioscience) and 1 × Brefeldin (eBioscience) in RPMI growth media for 4 h. After staining with various cell surface markers, fixed and permeabilized cells were stained with cytokine-specific antibodies as previously described (24). A 100 µL suspension of the stained cell population in FACS buffer was run on a BD Accuri C6 Plus flow cytometer (BD Biosciences, USA) and analyzed using FlowJo software.

### Cytokine immunoassay and HMGB1 measurement from the lung

From each pig, 0.5 grams of lung tissue was minced and placed in a Safe-Lock microtube containing a sterile steel bead. Then, 1 mL of T-PER Tissue Protein Extraction Buffer containing Halt protease and phosphatase inhibitor cocktail (ThermoFisher Scientific) was added to each tube and incubated for 5 minutes at room temperature. Subsequently, the tissues were homogenized using TissueLyser II (QIAGEN). The homogenates were centrifuged at 13,000 rpm for 10 min at 4°C. The protein concentration of the resulting supernatant was measured using the Pierce BCA Protein Assay Kit (ThermoFisher Scientific). The assay was performed according to the manufacturer’s instructions. All protein samples were diluted to 5 mg/mL with T-per buffer and used for cytokine immunoassay.

The cytokine levels (IFN-α, IFN-γ, IL-1β, IL-4, IL-6, IL-8, IL-10, IL-12p40, and TNF-α) in the lung homogenate were measured using a ProcartaPlex Porcine Cytokine & Chemokine Panel 1 9-Plex Immunoassay (ThermoFisher Scientific), according to the manufacturer’s instructions. The concentration of each cytokine was measured using a Luminex 200 system (Luminex Corporation, USA). In addition, HMGB1, one of the well-recognized proinflammatory factors enhancing inflammatory response (40), was measured from the lung homogenates with an HMGB1 ELISA kit (cat. No. SEA399Po; Cloud-Clone Corp.) according to the manufacturer’s instructions as previously described (41).

### mRNA quantification of immune regulators from BAL cells

Total RNA was extracted from 5 × 10^6^ BAL cells using 0.5 mL of NucleoZOL reagent and the NucleoSpin RNA Set for NucleoZOL (Macherey-Nagel, Germany), according to the manufacturer’s instructions. The extracted RNA was confirmed using a NanoDrop spectrophotometer (Biospec-nano, Shimadzu Scientific Instruments, Japan), followed by a Qubit 2.0 fluorometer using a Qubit RNA Broad Range (BR) assay kit for quantification. Subsequently, 5 µg of RNA was used for complementary DNA (cDNA) synthesis using a WizScript cDNA Synthesis Kit (Wizbiosolutions, Republic of Korea), according to the manufacturer’s instructions. Real-time PCR was performed on a 7500 Fast Real-time PCR system (Applied Biosystems, USA) with a Power SYBR Green PCR master mix kit (Applied Biosystems). Various chemokines (*CCL2*, *CCL5*, *CCL8,* and *CXCL10*) and their receptors (*CCR4*, *CCR5*, *CXCR5*) (28, 42, 43), interferon-stimulated genes (ISGs; *ISG15*, *ISG12(A*), *MX1,* and *MX2*) (44–47), immune checkpoint molecules (*PD1*, *PDL1*, *PDL2*, *CTLA4*, *CD200R1*, *LAG3*, *TIM3*, and *IDO1*) (20, 28, 29, 33), potential lung disease progression marker *SPP1* (48), and potential M2 macrophage marker *TREM2* (49, 50) were selected as target genes. The comparative cycle threshold (Ct) method (2^-ΔΔCt^) was used for relative quantification by normalizing target genes to the expression of the housekeeping gene porcine *HPRT1* (51). All qPCR results were confirmed by melting curve analysis, and the primer set sequences for the target and housekeeping genes are provided in Table S3.

### Measurement of serum kynurenine/tryptophan ratio and validation of IDO1 production

As high expression of IDO1 mRNA was detected from BAL cells, tryptophan catabolism and kynurenine production by Indoleamine 2,3-dioxygenase 1 (IDO1) through the kynurenine pathway (30, 31) were assessed by their concentration in serum from all groups. The serum kynurenine/tryptophan (Kyn/Trp) ratio was measured using the kynurenine/tryptophan ratio ELISA pack (cat. No. ISE-2227; ImmuSmol) according to the manufacturer’s instructions.

To validate IDO1 protein production in BAL cells, total protein was extracted from 5 × 10^6^ BAL cells of each pig. Cryopreserved BAL cells were thawed completely, and 1 mL of T-PER Tissue Protein Extraction Buffer containing Halt protease and phosphatase inhibitor cocktail (ThermoFisher Scientific) was added. After incubating for 5 min at room temperature, homogenates were centrifuged at 13,000 rpm for 10 min at 4°C. The protein concentration of the resulting supernatant was measured through the same protocol applied for lung protein mentioned above, and BAL cell protein samples were diluted to 1 mg/mL with T-per buffer. IDO1 protein concentrations were measured from both prepared lung proteins (5 mg/mL) and BALc cell protein (1 mg/mL) using Porcine Indoleamine 2, 3-Dioxygenase ELISA Kit (cat. No. MBS738204; MyBioSource) according to the manufacturer’s instructions.

### Statistics and correlation matrix analysis

All statistical analyses and data visualizations were performed using GraphPad Prism (v10.0.3, GraphPad Software) and R software (v4.3.1). Group comparisons for continuous variables were performed using one-way or two-way ANOVA, followed by Tukey’s post hoc test when appropriate. To capture temporal responses (e.g., viremia, nasal shedding, and antibody levels), area under the curve (AUC) analyses were conducted using the trapezoidal rule. Importantly, because half of the animals (*n* = 3 per group) were euthanized at 7 dpi for tissue and bronchoalveolar lavage (BAL) collection, AUCs were calculated over two separate intervals: 0–7 dpi (*n* = 6) based on full cohort, allowing robust comparisons during the acute infection phase; 0–14 dpi (*n* = 3) based on surviving animals, providing insight into the progression phase. Two-way ANOVA with post hoc tests was used to evaluate group differences across these time intervals. Tissue viral loads and immune parameters (e.g., cytokines, mRNA expression, flow cytometry) were analyzed separately at 7 dpi and 14 dpi, using data from independently euthanized animals (*n* = 3 per time point) and thus were not subjected to longitudinal paired analysis. To explore associations among clinical and immunological variables (e.g., viremia, ADWG, cytokine expression, kynurenine pathway markers), a Spearman correlation matrix was generated using the "corrplot" package in R. Correlations with *P* < 0.01 were considered statistically significant.

## RESULTS

### Clinical outcome

**Fig 1:**
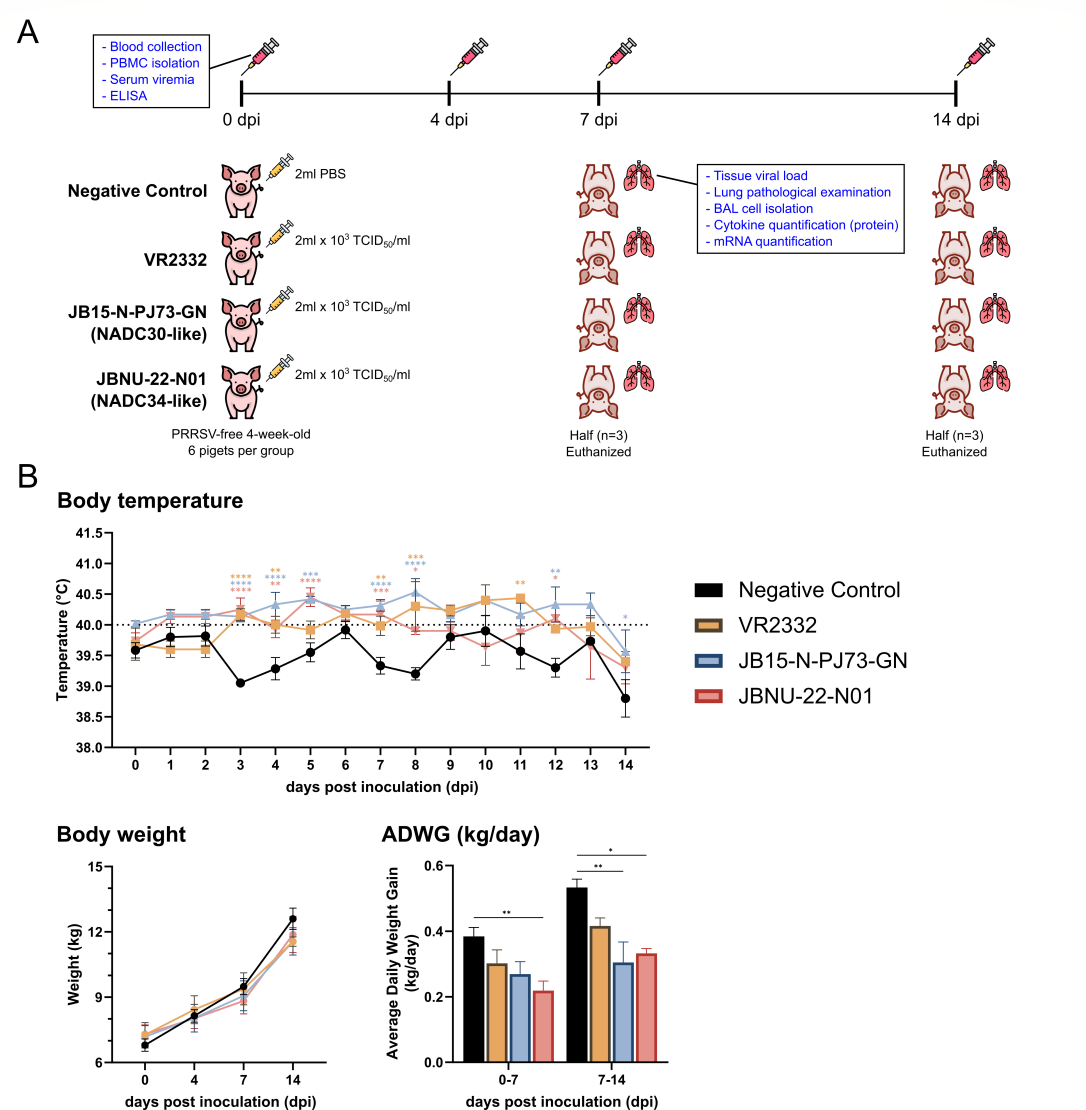
Animal study design and clinical outcome. (**A**) Overview of the animal study. (**B**) Clinical outcomes due to PRRSV-2 infections, including body temperature, changes in body weight, and average daily weight gain (ADWG). Data are shown as mean ± SEM, and asterisks (*) represent values that differ significantly from the negative control group (* indicates *P*-values < 0.05, ** indicates *P*-values < 0.01, *** indicates *P*-values < 0.001, and **** indicates *P*-values < 0.0001).

A total of 24 4-week-old piglets were purchased from a PRRSV-negative farm and subjected to a virus challenge experiment to investigate virus pathogenicity and virus-induced immune responses (Fig. 1A). All PRRSV-infected groups, except the negative control, exhibited high fevers beginning at 3 dpi, which persisted until 8 dpi. The JBNU-22-N01 group showed a statistically significant reduction in average daily weight gain (ADWG) compared with the control group from 0 to 7 dpi (*P* < 0.01). From 7 to 14 dpi, both the JBNU-22-N01 (*P* < 0.05) and PJ73 (*P* < 0.01) groups showed significantly reduced ADWG compared with the control group (Fig. 1B). Importantly, none of the JBNU-22-N01-infected animals exhibited severe clinical symptoms or mortality throughout the study.

### Serum viremia and antibody titer

**Fig 2:**
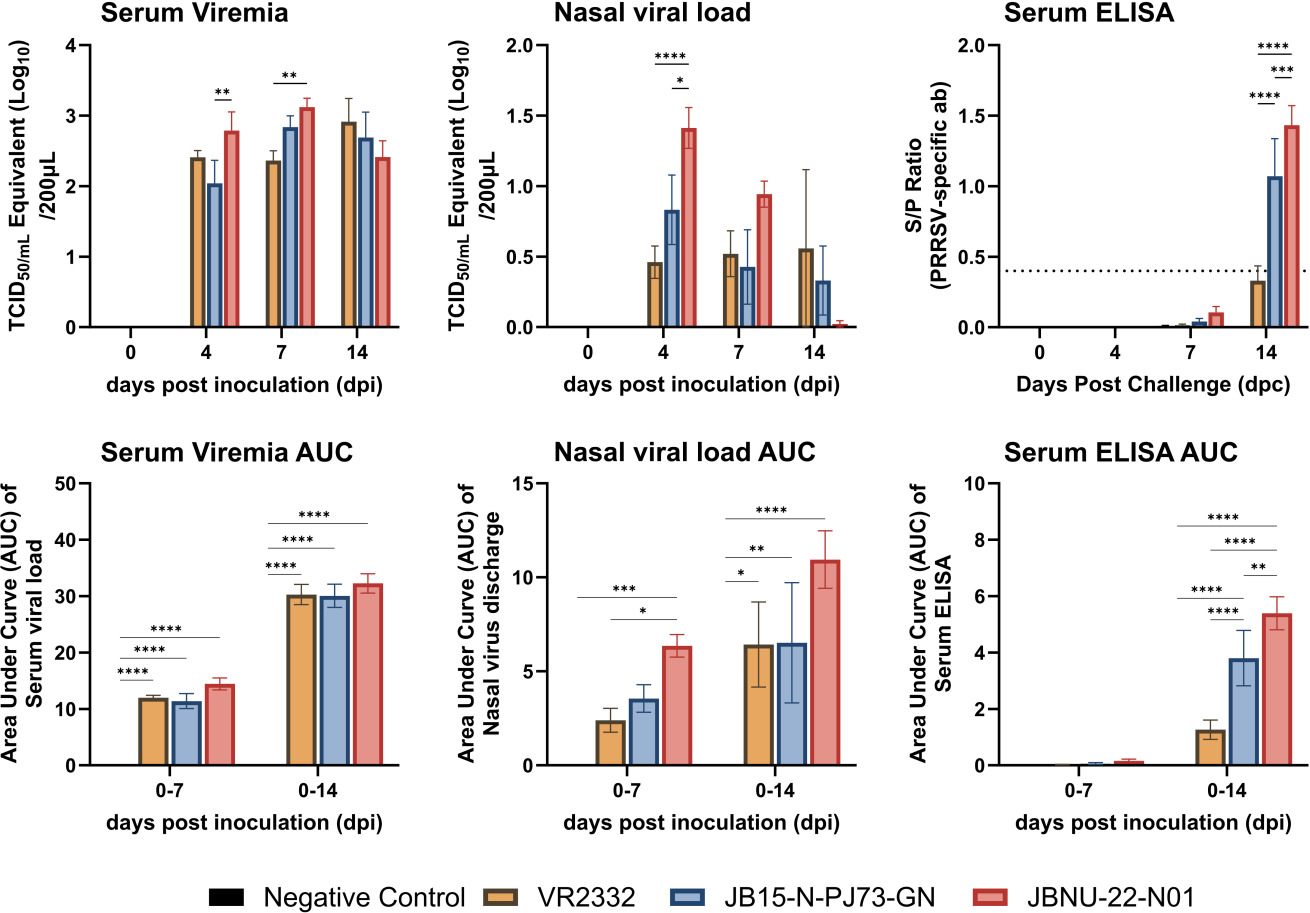
Serum viremia, nasal virus shedding, and PRRSV-specific antibody titers. The viral loads in sera and nasal swabs obtained from negative control and PRRSV-2-infected pigs at 0, 4, 7, and 14 dpi were quantified by qRT-PCR. The viral titers were calculated based on a standard curve of the threshold cycle number plotted against the known virus titer. PRRSV-specific IgG in sera was quantified using the conventional ELISA kit at the same dpi. Lower panels show AUC (area under the curve) values calculated using the trapezoidal rule for the intervals 0–7 dpi (*n* = 6) and 0–14 dpi (*n* = 3), reflecting cumulative virus replication and antibody response. AUC-based comparisons were analyzed using two-way ANOVA with post hoc testing. Data are shown as mean ± SEM, and asterisks (*) represent values that differ significantly from each other (* indicates *P*-values < 0.05, ** indicates *P*-values < 0.01, *** indicates *P*-values < 0.001, and **** indicates *P*-values < 0.0001).

Serum viremia was significantly higher in the JBNU-22-N01 group compared with the PJ73 and VR2332 groups at 4 and 7 dpi (*P* < 0.01), with no significant differences observed at 14 dpi. For nasal viral shedding, the JBNU-22-N01 group displayed significantly higher levels than the VR2332 group (*P* < 0.0001) and the PJ73 group (*P* < 0.05) at 4 dpi, and the trend continued through 7 dpi. PRRSV-specific antibody levels rose more rapidly in the PJ73 and JBNU-22-N01 groups than in the VR2332 group (Fig. 2).

To evaluate overall differences across the infection period, area under the curve (AUC) values were calculated using the trapezoidal rule for two time intervals: 0–7 dpi (*n* = 6) and 0–14 dpi (*n* = 3). These AUC values were analyzed using two-way ANOVA with post hoc tests to assess the effects of viral strain and time. Significant differences were observed in AUC values, with JBNU-22-N01 showing higher cumulative nasal shedding during the 0–7 dpi interval and higher antibody titers during the 0–14 dpi interval (Fig. 2, lower panels).

### Tissue viral loads

**Fig 3:**
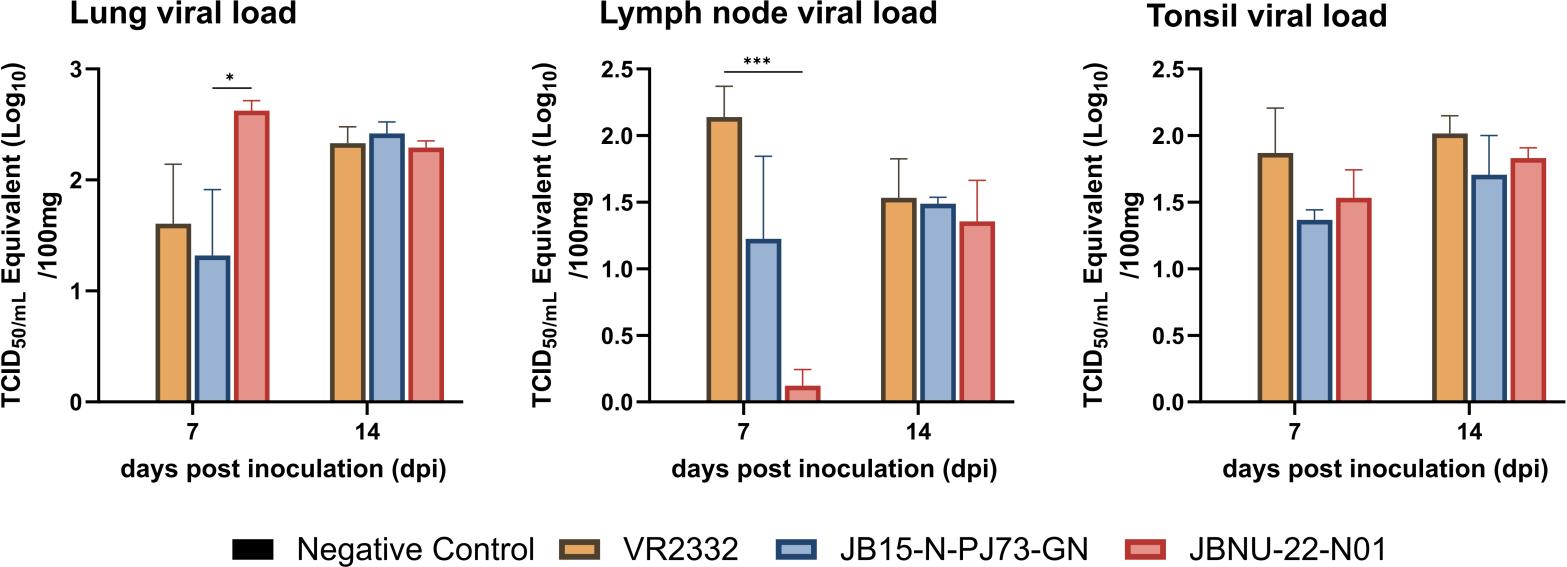
Viral loads in tissues. The viral loads in the lung, bronchial lymph node (BLN), and tonsil obtained from necropsied pigs at 7 and 14 dpi were quantified by qRT-PCR. The viral titers were calculated based on a standard curve of the threshold cycle number plotted against the known virus titer. Data are shown as mean ± SEM, and asterisks (*) represent values that differ significantly from each other (* indicates *P*-values < 0.05 and *** indicates *P*-values < 0.001).

For tissue samples, the JBNU-22-N01 group showed the highest lung viral load at 7 dpi, significantly greater than PJ73 (*P* < 0.05). At 14 dpi, lung viral loads among all strains were similar. In the bronchial lymph node (BLN), the JBNU-22-N01 group had the lowest viral load at 7 dpi, whereas the VR2332 group had the highest, significantly exceeding JBNU-22-N01 (*P* < 0.001). At 14 dpi, BLN viral loads were comparable across groups. No significant differences in tonsil viral loads were noted among groups at either 7 or 14 dpi (Fig. 3).

### Lung pathology

**Fig 4:**
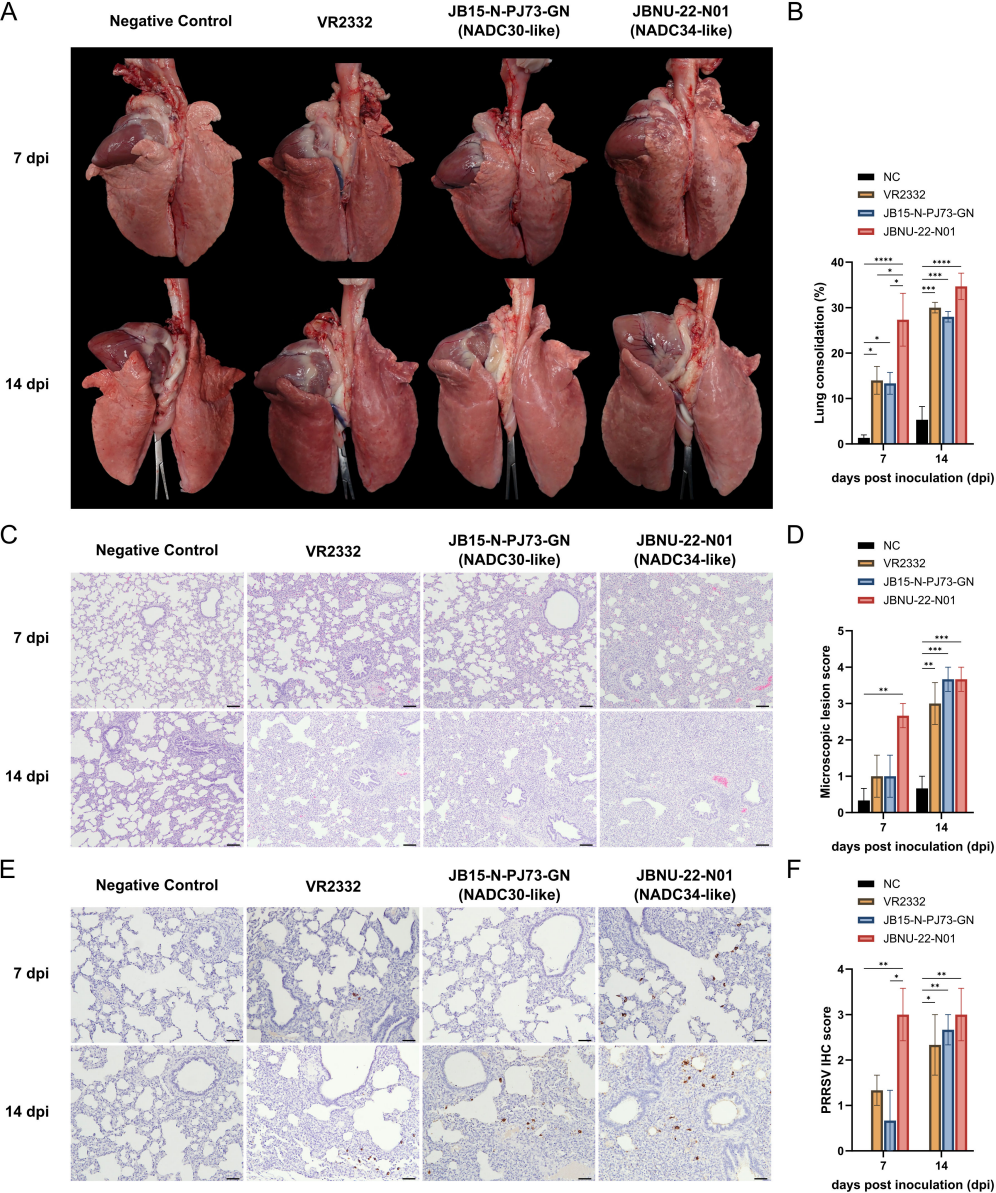
Overview of lung pathology. The representative (**A**) gross lesion, (**C**) microscopic lesion, and (**E**) IHC staining of necropsied pigs at 7 and 14 dpi are presented with (**B**) macroscopic lesion score, (**D**) microscopic lesion score, and (**F**) IHC score. Data are shown as mean ± SEM, and asterisks (*) represent values that differ significantly from each other (* indicates *P*-values < 0.05, ** indicates *P*-values < 0.01, *** indicates *P*-values < 0.001, and **** indicates *P*-values < 0.0001).

Consistent with lung viral load data, JBNU-22-N01-infected lungs displayed significantly more extensive gross lesions than other infection groups (*P* < 0.05) at 7 dpi. By 14 dpi, all infected groups showed similar lesion severity (Fig. 4A and B). At 7 dpi, JBNU-22-N01-infected lungs also exhibited more severe interstitial pneumonia with alveolar wall thickening, due to type 2 pneumocyte proliferation and inflammatory cell infiltration, compared with other groups. Mild-to-moderate necrotic cell presence in alveoli was noted in JBNU-22-N01-infected lungs at 7 dpi, which was also observed in VR2332- and PJ73-infected lungs at 14 dpi (Table S1). Microscopic lesion scores were similar across groups by 14 dpi (Fig. 4C and D). IHC revealed significantly higher PRRSV antigen levels in JBNU-22-N01-infected lungs than those of the PJ73 group (*P* < 0.05) and VR2332 group at 7 dpi, although antigen levels were comparable across groups by 14 dpi (Fig. 4E and F).

### Flow cytometry of BAL cells

**Fig 5:**
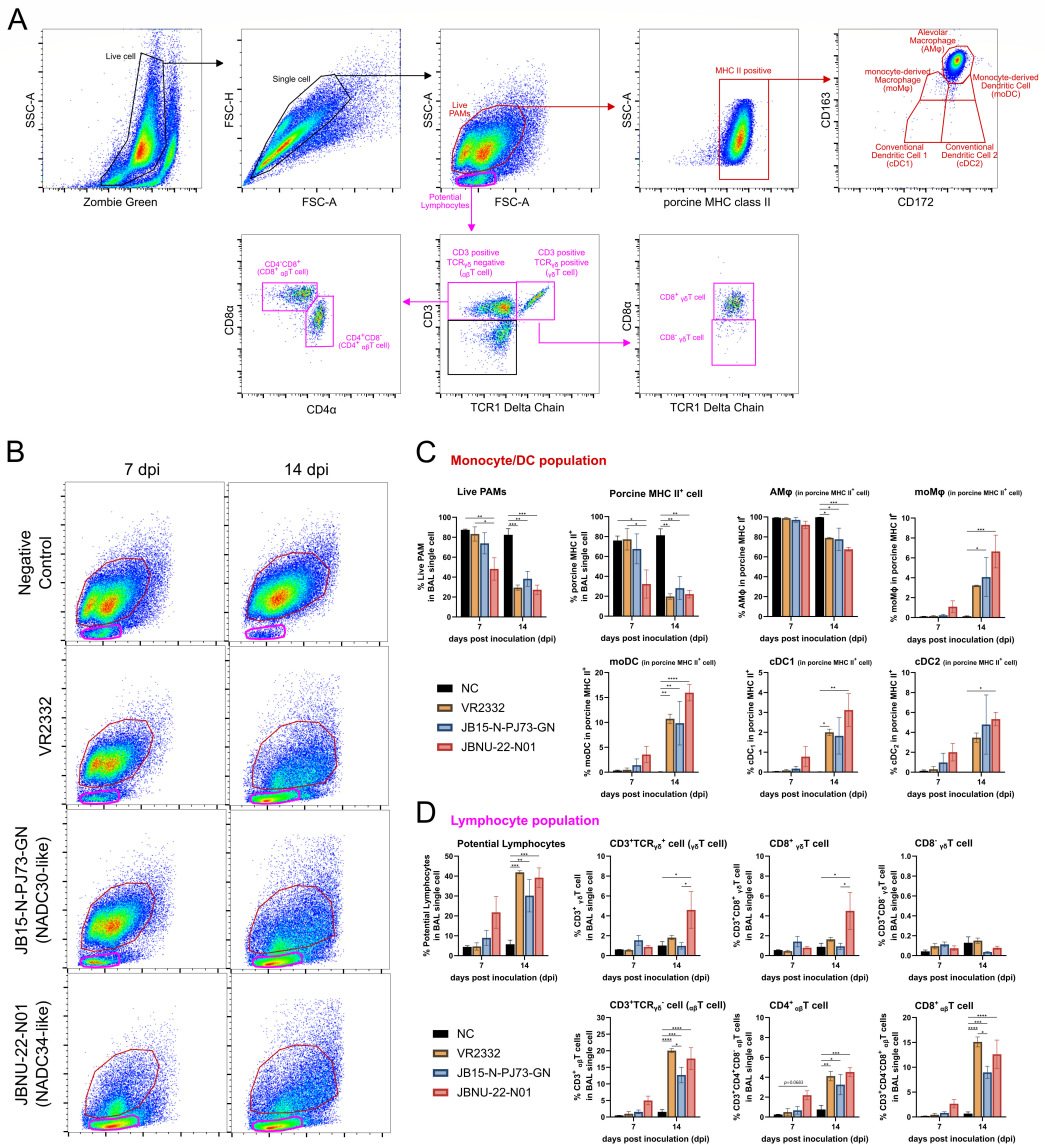
Fluctuations in the frequencies of monocyte/dendritic cells and infiltrated T cells in the lung. Single-cell suspensions of BAL cells obtained from the right diaphragmatic and cardiac lobes of necropsied pigs at 7 and 14 dpi were stained and measured by flow cytometry. (**A**) Gating strategy for macrophage/dendritic cell (DC) network (red) and infiltrated T lymphocytes (pink) in the lung. (**B**) Representative FSC-A/SSC-A dot plot showing fluctuations of live PAMs (red circle) and potential lymphocytes (pink circle) in the lung of necropsied pigs. (**C**) Fluctuations in the macrophage/DC network within lung including live PAMs, MHCII^+^ cells, MHCII^+^/CD172α^+^/CD163^high^ (alveolar macrophages; AMɸ), MHCII^+^/CD172α^+^/CD163^int^ (monocyte-derived macrophages; moMɸ), MHCII^+^/CD172α^+^/CD163^low^ (monocyte-derived dendritic cells; moDC), MHCII^+^/CD172α^+^/CD163^-^ (conventional dendritic cells 2; cDC2), and MHCII^+^/CD172α^-^_/_CD163^-^ (conventional dendritic cells 1; cDC1). (**D**) Fluctuation in the infiltrated T lymphocytes within lung including CD3^+^/TCRγδ^-^ (αβ T) cells, D3^+^/TCRγδ^-^/CD4^+^/CD8^-^ cell (CD4^+^ αβ T), CD3^+^/TCRγδ^-^/CD4^-^/CD8^+^ (CD8^+^ αβ T) cell, CD3^+^/TCRγδ^+^ (γδ T) cells, CD3^+^/TCRγδ^+^/CD8^-^ (CD8^-^ γδT; proinflammatory γδ T cells), and CD3^+^/TCRγδ^+^/CD8^+^ (CD8^+^ γδ T; regulatory γδ T cells). Data are shown as mean ± SEM, and asterisks (*) represent values that differ significantly from each other (* indicates *P*-values < 0.05, ** indicates *P*-values < 0.01, *** indicates *P*-values < 0.001, and **** indicates *P*-values < 0.0001).

Flow cytometry analysis of BAL cells at 7 and 14 dpi revealed dynamic changes in immune cell populations during infections (Fig. 5A). At 7 dpi, the JBNU-22-N01 group exhibited a more rapid decline in the frequency of pre-existing PAMs, accompanied by significant lymphocyte infiltration into the lung. This trend was observed across all infection groups by 14 dpi, albeit with varying magnitudes (Fig. 5B). In the macrophage and dendritic cell (DC) populations, JBNU-22-N01 infection was associated with accelerated monocyte-derived macrophages (moMɸ) and distinct dendritic cell subsets (moDC, cDC1, and cDC2) as early as 7 dpi. These changes were also present in other infection groups by 14 dpi (Fig. 5C).

In parallel, αβ T cell populations, including CD3^+^, CD4^+^, and CD8^+^ αβ T cells, demonstrated more rapid expansion in JBNU-22-N01-infected lungs at 7 dpi compared with other groups. By 14 dpi, all infection groups exhibited substantial increases in these populations; however, a marked elevation in CD8^+^ γδ T cells (regulatory γδ T cells) was uniquely observed in the JBNU-22-N01 group (Fig. 5D). Notably, at 14 dpi, CD8^+^ αβ T cells displayed a more pronounced infiltration (9-15%) compared with CD4^+^ αβ T cells (2%–5%) across all infections, highlighting a differential recruitment of effector T cells during pulmonary immune responses.

**Fig 6:**
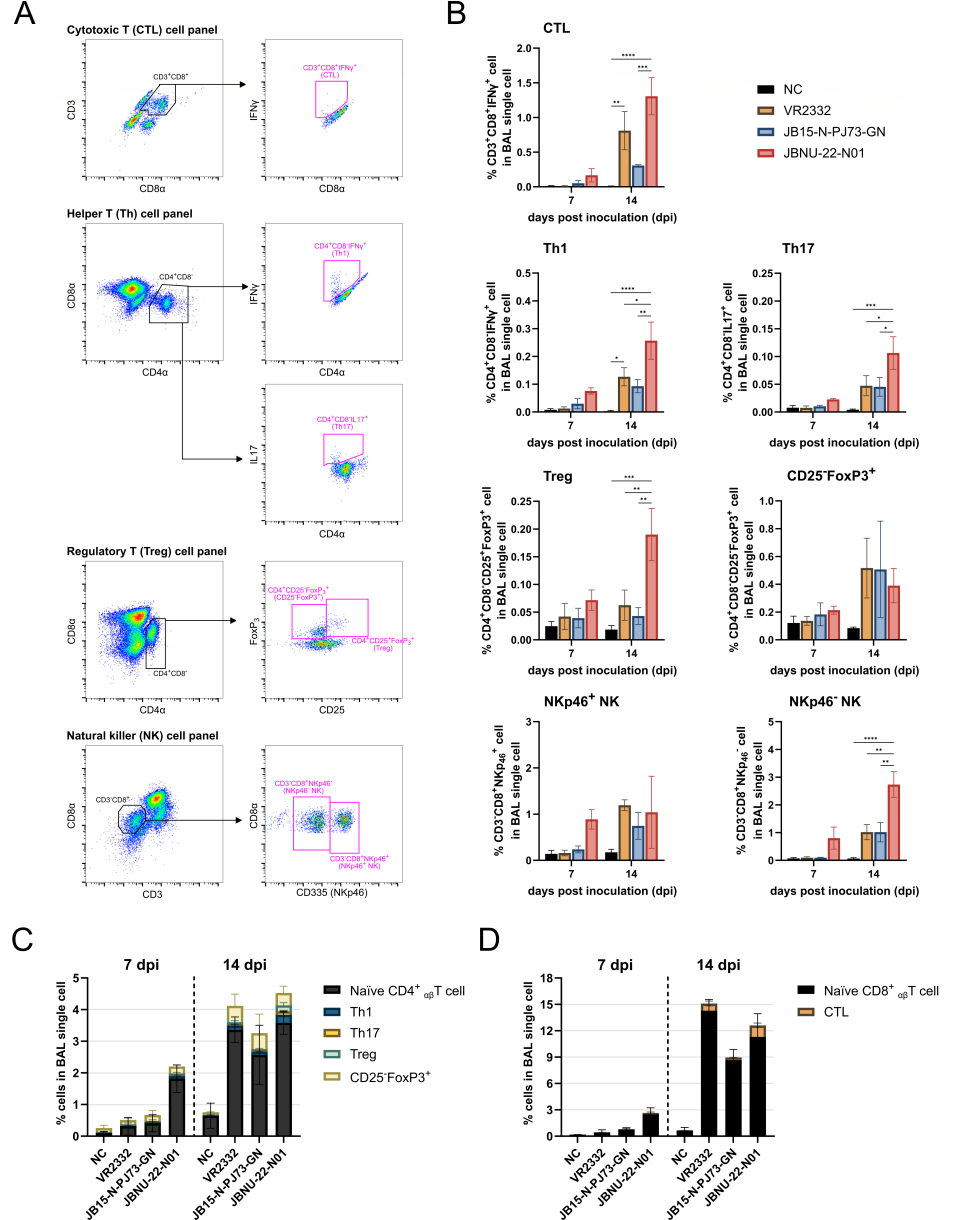
Frequencies of T cell subsets and NK cells in the lung. BAL cells were stained to measure T cell subsets and NK cells by flow cytometry. (**A**) Gating strategy. (**B**) Fluctuations in the infiltrated NK cells (CD3^-^/CD8^+^/NKp46^+^; NKp46^+^ NK, CD3^-^/CD8^+^/NKp46^-^; NKp46^-^ NK) and T cell subsets (CD3^+^/CD8^+^/IFNγ^+^; CTL, CD4^+^/CD8^-^/IFNγ^+^; Th1, CD4^+^/CD8^-^/IL17^+^; Th17, CD4^+^/CD8^-^/CD25^+^/FoxP3^+^; Treg and CD4^+^/CD8^-^/CD25^-^/FoxP3^+^; CD25^-^FoxP3^+^ cell) within lung. (**C**) The proportions of CD4^+^ αβ T cell subsets (Th1, Th17, and FoxP3^+^ cells) within infiltrated CD4^+^ αβ T cells. (**D**) The proportions of CD8^+^ αβ T cell subset (CTL) within infiltrated CD8^+^ αβ T cells. Data are shown as mean ± SEM, and asterisks (*) represent values that differ significantly from each other (* indicates *P*-values < 0.05, ** indicates *P*-values < 0.01, *** indicates *P*-values < 0.001, and **** indicates *P*-values < 0.0001).

As significant infiltration of CD4^+^ and CD8^+^ αβ T cell populations was detected from BAL cells, CD8^+^ effector T cell subsets, specifically cytotoxic T lymphocytes (CTLs; CD3^+^/CD8^+^/IFNγ^+^), and CD4^+^ effector T cell subsets, including T helper 1 (Th1; CD4^+^/CD8^-^/IFNγ^+^) and 17 (Th17; CD4^+^/CD8^-^/IL17^+^) cells, were analyzed along with regulatory CD4^+^ T cell subsets (Tregs; CD4^+^/CD8^-^/CD25^+^/FoxP3^+^ and CD25^-^/FoxP3^+^ cells; CD4^+^/CD8^-^/CD25^-^/FoxP3^+^). Additionally, NK cells were examined, including NKp46^+^ NK (CD3^-^/CD8^+^/NKp46^+^) and NKp46^-^ NK (CD3^-^/CD8^+^/NKp46^-^) subtypes (Fig. 6A).

At 7 dpi, the JBNU-22-N01 group exhibited rapid expansion of CTLs, Th1, Th17, and NK cells, corresponding with an overall increase in αβ T cells. By 14 dpi, T cell subsets, including CTLs, Th1, Th17, Tregs, and NKp46^-^ NK cells, were significantly elevated in the JBNU-22-N01 group compared with other groups (Fig. 6B). Notably, among all infections, FoxP3^+^ T cells (Tregs and CD25^-^FoxP3^+^ cells) showed a higher proportion (0.5%–1%) than effector CD4^+^ T cells (Th1 and Th17; <0.5%) relative to the overall CD4^+^ αβ T cell population (3%–5%) at 14 dpi (Fig. 6C). Similarly, although the proportion of CD8^+^ αβ T cells reached 9%–15% at 14 dpi, CTLs represented only a small fraction (<1.5%) of this population (Fig. 6D).

### Flow cytometry of PBMC

**Fig 7:**
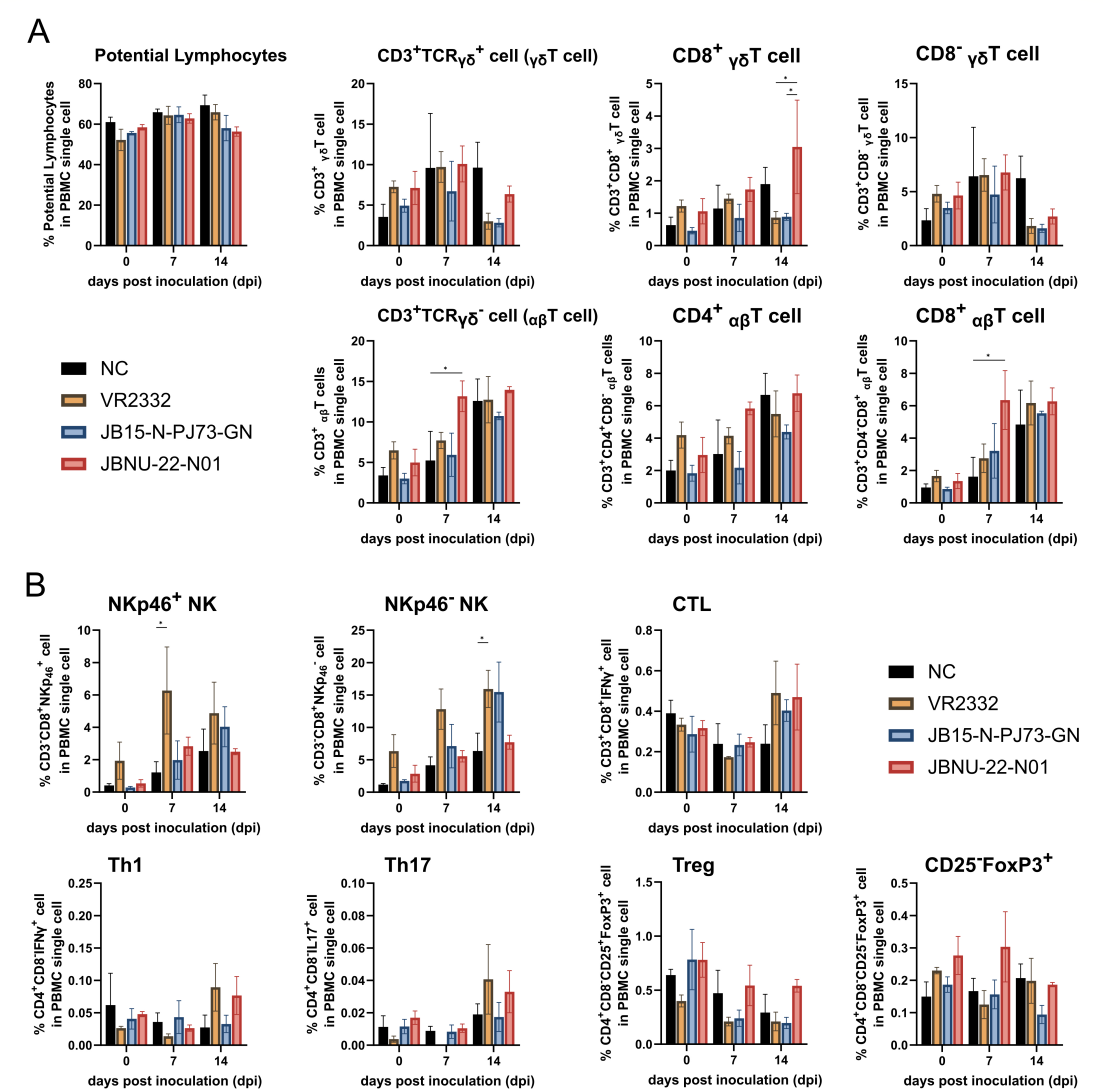
Fluctuations in the frequencies of T cells and NK cells in PBMC. Single-cell suspensions of PBMC collected from whole blood collected at 0, 7, and 14 dpi were stained for T cells/subsets and NK cells. Gating strategy was the same as BAL cells (Fig. 5 and 6)(. (**A**) Fluctuation in the general T cell population (αβT, CD4^+^ αβ T, CD8^+^ αβt, γδT, CD8^-^ γδT, and CD8^+^ γδt) within PBMC. (**B**) Fluctuation in the NK cells (NKp46^+^ NK and NKp46^-^ NK) and T cell subsets (CTL, Th1, Th17, Treg, and CD4^+^/CD8^-^/CD25^-^/FoxP3^+^ cell) within PBMC. Data are shown as mean ± SEM, and asterisks (*) represent values that differ significantly from each other (* indicates *P*-values < 0.05).

In peripheral blood mononuclear cells (PBMCs), no significant increase in T cell populations was observed until 14 dpi, consistent with prior studies showing a delayed peripheral T cell response post-PRRSV infection (24). The exceptions were a significant increase in CD3^+^ and CD8^+^ αβT cells in the JBNU-22-N01 group at 7 dpi, relative to the negative control (*P* < 0.05), and CD8+ γδT cells in the JBNU-22-N01 group at 14 dpi compared with other infection groups (*P* < 0.05) (Fig. 7A). Among T cell subsets and NK cells, only NKp46^+^ NK and NKp46^-^ NK cells were significantly elevated in the VR2332 group at 7 and 14 dpi, respectively, compared with the control group (*P* < 0.05) (Fig. 7B).

**Fig 8:**
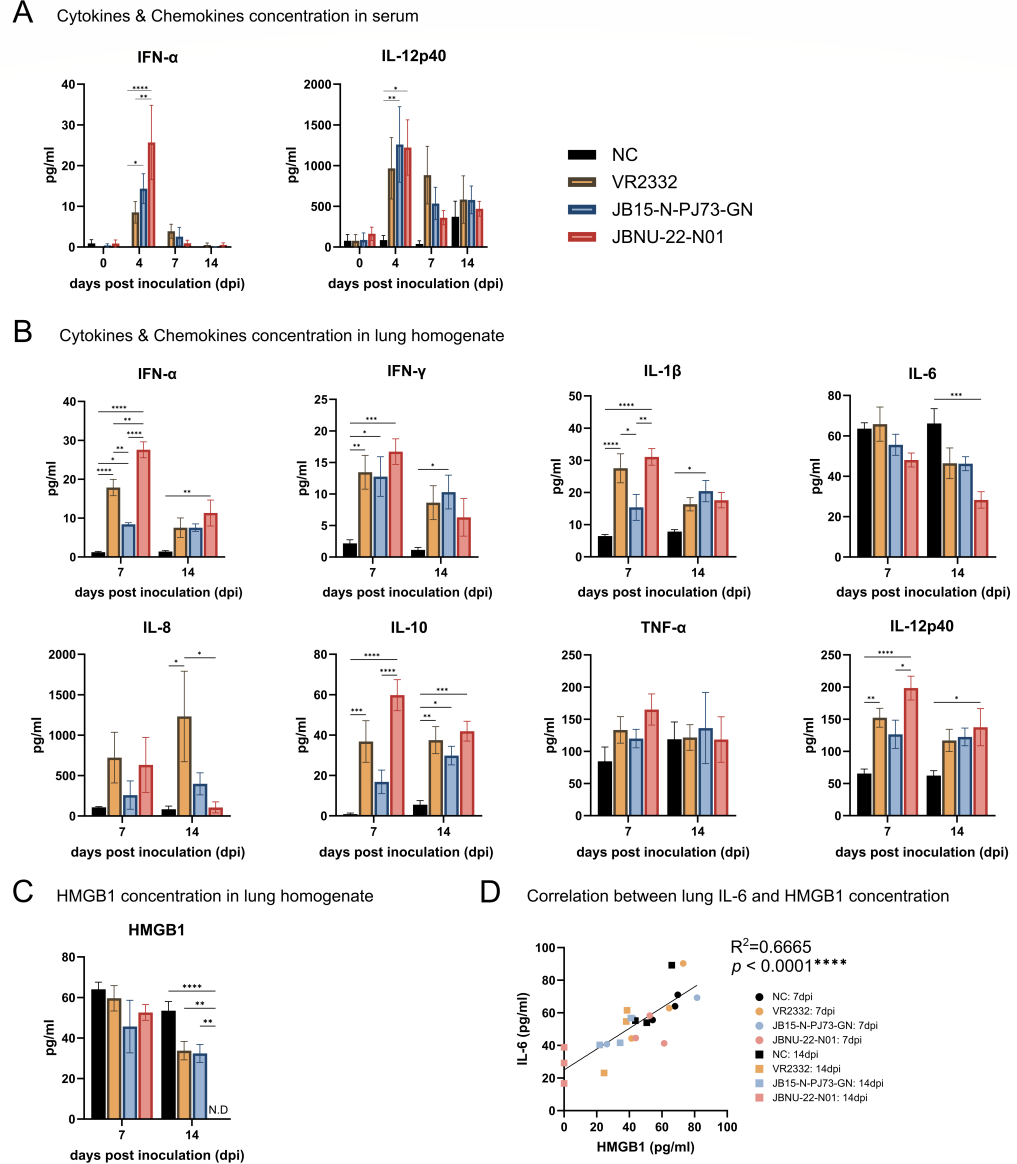
Fluctuations of cytokine/chemokine and HMGB1 in the lung. (**A**) The protein concentrations of cytokines IFN-α and IL-12p40 in the serum. (**B**) The protein concentrations of cytokines/chemokines (IFN-α, IFN-γ, IL-1β, IL-6, IL-8, IL-10, IL-12p40, and TNF-α) in the lung homogenates. (**C**) The HMGB1 concentration in the lung homogenates. (**D**) Spearman correlation between lung IL-6 and HMGB1. Data are shown as mean ± SEM, and asterisks (*) represent values that differ significantly from each other (* indicates *P*-values < 0.05, ** indicates *P*-values < 0.01, *** indicates *P*-values < 0.001, and **** indicates *P*-values < 0.0001).

### Systemic and local cytokine reactions

The protein levels of cytokines were measured by bead-based immunoassay from the serum and lung homogenates. In serum, only IFN-α and IL-12p40 could be detected, whereas other cytokines were under the detection level. Both cytokines were increased in all infection groups at 4 dpi and gradually decreased. The JBNU-22-N01-infected group showed a significant increase in serum IFN-α at 4 dpi compared with other groups, especially with VR2332 infection (*P* < 0.01) (Fig. 8A).

In the lung homogenates, the JBNU-22-N01 group displayed the highest increase in proinflammatory cytokines (IFN-α, IFN-γ, IL-1β, and IL-12p40) and the regulatory cytokine IL-10 at 7 dpi. The VR2332 infected group also showed a significant increase in IFN-α, IFN-γ, IL-1β, IL-12p40, and IL-10 compared with the negative control group at 7 dpi, but the levels were lower than the JBNU-22-N01 group. PJ73 showed a significant increase only in IFN-α and IFN-γ at 7 dpi compared with the negative control, but showed a significant increase in IFN-γ and IL-1β compared with the control at 14 dpi. At 14 dpi, all virus infections showed a similar trend, whereas the JBNU-22-N01-infected group showed a sustained significant increase in IFN-α and IL-12p40 compared with a negative control group. Notably, proinflammatory cytokine IL-6 gradually decreased among all infections until 14 dpi, in which JBNU-22-N01 showed a significant decrease compared with the negative control group at 14 dpi (*P* < 0.001) (Fig. 8B).

Extracellular HMGB1 is known to activate NF-κB and interferon regulatory factor (IRF)-mediated signaling pathways, inducing the expression of proinflammatory cytokines and chemokines, including TNF-α, IL-1β, IFN-γ, IL-6, and IL-8 (40, 48). In a previous study, HP-PRRSV infection resulted in upregulation of HMGB1 and IL-6, with a significant positive correlation between the two molecules (41). In contrast, in this study, HMGB1 protein levels gradually declined in all PRRSV-infected groups until 14 dpi, mirroring the trend observed for IL-6. Notably, HMGB1 levels in the JBNU-22-N01 group dropped below the detection limit (Fig. 8C). A strong positive correlation between IL-6 and HMGB1 was observed (*P* < 0.0001) (Fig. 8D).

### mRNA expressions of chemokines, their receptors, ISGs, and immune checkpoint molecules in BAL cells

**Fig 9:**
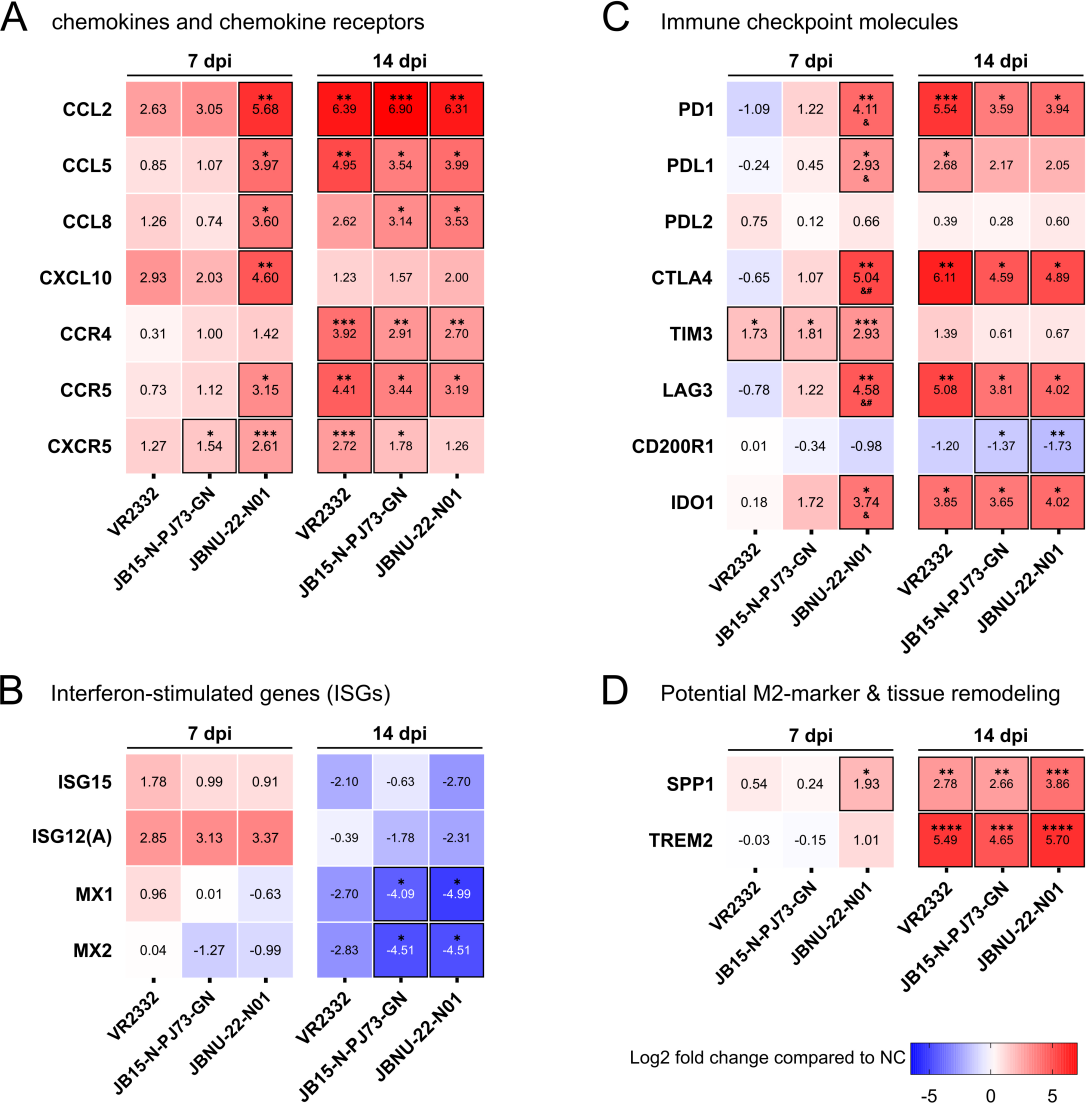
mRNA expressions in BAL cells. (**A**) Chemokine (*CCL2*, *CCL5*, *CCL8*, and *CXCL10*) and chemokine receptor (*CCR4*, *CCR5*, and *CXCR5*) mRNA levels. (**B**) Interferon-stimulated gene (*ISG15*, *ISG12(A*), *MX1*, and *MX2*) mRNA levels. (**C**) Immune checkpoint molecule (*PD1*, *PDL1*, *PDL2*, *CTLA4*, *CD200R1*, *LAG3*, *TIM3*, and *IDO1*) mRNA levels. (**D**) mRNA levels of lung disease progression marker *SPP1* and M2 macrophage marker *TREM2*. The mRNA expression levels were expressed as log2 fold change compared with the negative control. Data are shown as mean value, and asterisks (*) represent values that differ significantly from the negative control (* indicates *P*-values < 0.05, ** indicates *P*-values < 0.01, *** indicates *P*-values < 0.001, and **** indicates *P*-values < 0.0001). Statistically significant differences (*P*-values < 0.05) between infection groups are marked with ampersand (&, VR2332 with JBNU-22-N01) and hash (#, JB15-N-PJ73-GN with JBNU-22-N01).

When measuring mRNA expression levels from BAL cells, chemokines CCL2, *CCL5*, *CCL8,* and CXCL10 and receptors CCR5 and CXCR5 were significantly upregulated only in JBNU-22-N01 infection at 7 dpi compared with the negative control. However, all chemokines, except CXCL10, and receptors were significantly upregulated in all infection groups at 14 dpi (Fig. 9A). Among ISGs, only ISG15 and ISG12(A) were upregulated in all infections at 7 dpi. All ISGs were downregulated in all infection groups at 14 dpi, with significant downregulation of MX1 and MX2 in the PJ73 group and JBNU-22-N01 group (Fig. 9B). Immune checkpoint molecules including *PD1*, *PDL1*, *CTLA4*, *LAG3*, and *IDO1* were significantly upregulated in the JBNU-22-N01-infected group compared with the negative control and VR2332 infection at 7 dpi. *TIM3* was significantly upregulated in all infection groups at 7 dpi. At 14 dpi, all infection groups showed almost identical upregulated mRNA expression profiles of *PD1*, *PDL1*, *CTLA4*, *LAG3,* and *IDO1* compared with the negative control (Fig. 9C; Fig. S1). The gene expression of *SPP1* was significantly upregulated in JBNU-22-N01 infection at 7 dpi and in all infections at 14 dpi. *TREM2* gene was significantly upregulated in all infections at 14 dpi compared with the negative control, showing the highest significance (*P* < 0.0001) among all investigated genes (Fig. 9D).

### Increased serum Kyn/Trp ratio and IDO1 protein in BAL cells

**Fig 10:**
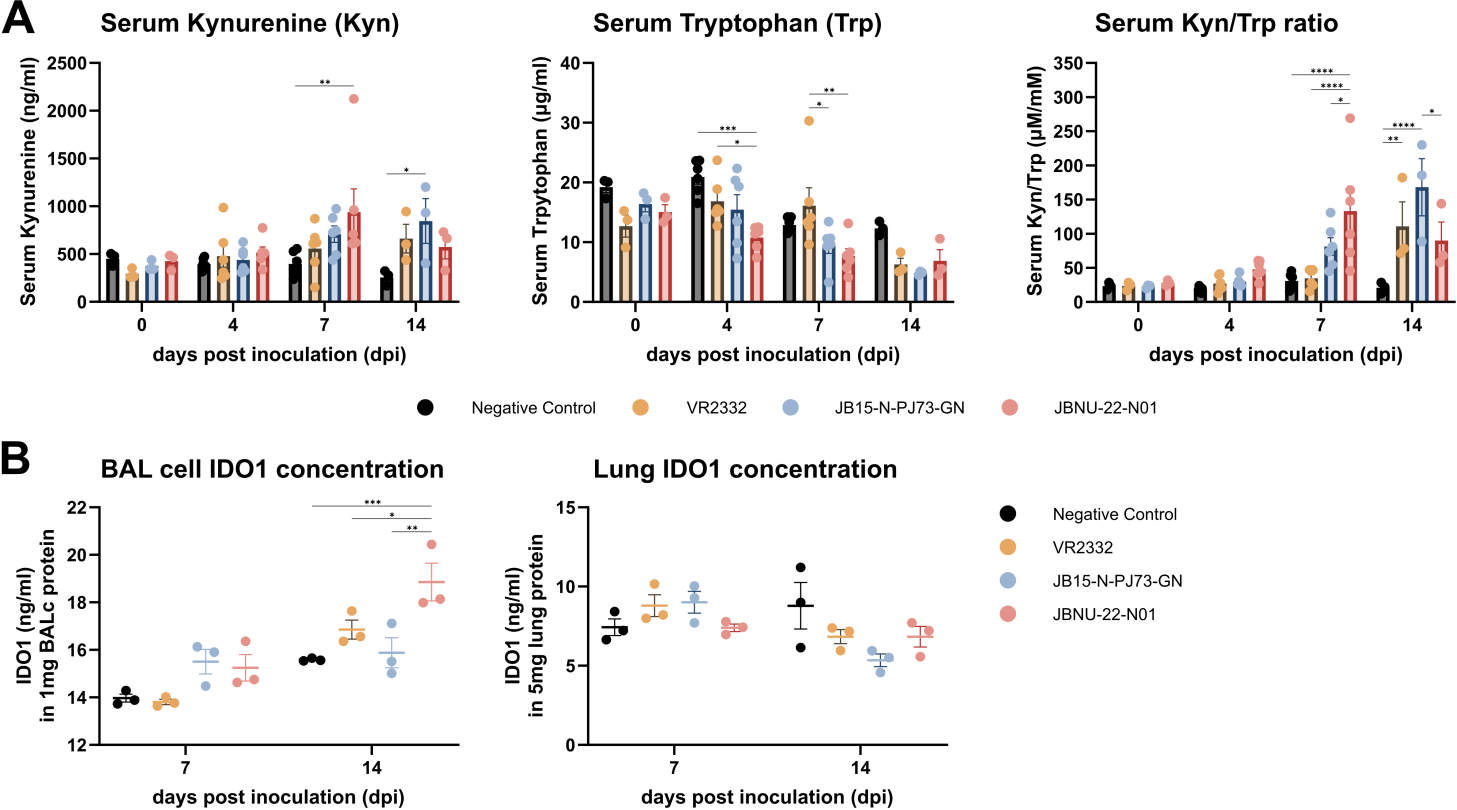
Serum Kyn/Trp ratio and IDO1 protein levels in BAL cells and lung tissues. (**A**) Kynurenine (Kyn) and tryptophan (Trp) concentrations, along with the kynurenine/tryptophan (Kyn/Trp) ratio in serum, were measured in PRRSV-infected pigs across 4, 7, and 14 dpi. (**B**) Protein levels of IDO1 were quantified from bronchoalveolar lavage (BAL) cells and lung tissue at 7 and 14 dpi. Data are represented as mean ± SEM, with statistical significance denoted as follows: * indicates *P*-values < 0.05, ** indicates *P*-values < 0.01, *** indicates *P*-values < 0.001, and **** indicates *P*-values < 0.0001.

The temporal changes in serum kynurenine (Kyn), tryptophan (Trp) concentrations, and the Kyn/Trp ratio, along with IDO1 concentrations in BAL cells and lung tissue following PRRSV infection, are presented in Fig. 10. The selection of these parameters was based on the well-established role of IDO1 in the kynurenine pathway of tryptophan metabolism, which is known to regulate immune responses and promote immunosuppression (31, 52, 53). This pathway has been extensively characterized in cancer and infectious disease models (52–55), and its activation in PRRSV-infected piglets suggests a potential role in virus-induced immune modulation.

In serum, Kyn levels were significantly elevated in the JBNU-22-N01 group and the PJ73 group compared with the negative control at 7 dpi (*P* < 0.01) and 14 dpi (*P* < 0.05), respectively. Conversely, Trp concentrations were gradually reduced from 4 to 14 dpi across infected groups, particularly in JBNU-22-N01 at 4 dpi. The serum Kyn/Trp ratio, an indicator of IDO1 activity with tryptophan catabolism, was elevated in all infected groups compared with controls. The JBNU-22-N01 group exhibited the highest increase at 7 dpi, whereas the PJ73 group peaked at 14 dpi (Fig. 10A), suggesting robust activation of the kynurenine pathway in response to infection. Analysis of IDO1 protein concentrations in BAL cells revealed significant upregulation in all infected groups, especially JBNU-22-N01, indicating enhanced immunosuppressive activity via the kynurenine pathway. BAL cell IDO1 levels were higher than the negative control group at both 7 dpi (PJ73 and JBNU-22-N01) and 14 dpi (VR2332 and JBNU-22-N01), with a notable significant increase in the JBNU-22-N01 group compared with others at 14 dpi. However, IDO1 levels in lung tissue did not show significant differences across infection groups (Fig. 10B).

Although a modest increase in serum Kyn/Trp ratio was observed in PRRSV-infected pigs, particularly in JB15 and JBNU-22-N01 groups, this trend was not paralleled by statistically significant changes in IDO1 protein levels in BAL or lung tissues at 7 dpi. Notably, the JBNU group showed relatively higher IDO1 protein expression in BAL at 14 dpi, but its serum Kyn/Trp ratio was lower than that of JB15. These discrepancies may reflect complex temporal and tissue-specific dynamics between local IDO1 expression and systemic tryptophan metabolism.

### Correlation matrix analysis

**Fig 11:**
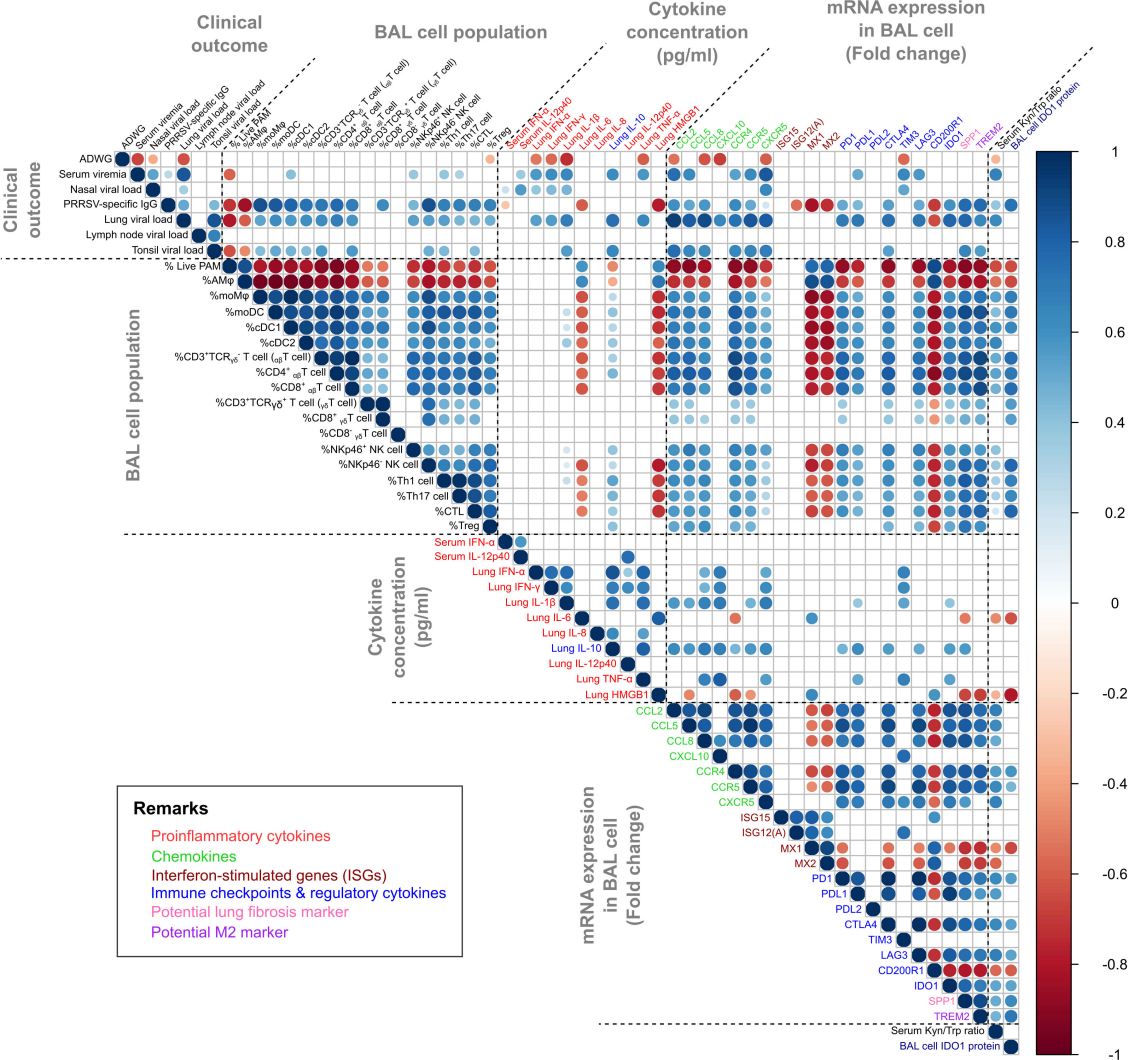
Correlation matrix analysis of clinical, immunological, and molecular parameters in PRRSV-infected pigs. Spearman correlation matrix displaying the relationships among clinical outcomes (e.g., ADWG, viremia), immune cell populations in BAL samples, cytokine levels in lung homogenates, mRNA expressions in BAL cells, and potential biomarkers of PRRSV-induced immunomodulation, such as serum kynurenine/tryptophan (Kyn/Trp) ratio. The Spearman correlation matrix was generated using the corrplot package in R software, with correlations considered significant at *P* < 0.01. Only significant correlations are indicated by circles, with non-significant correlations left blank. The color and size of the circles correspond to the level of correlation, ranging from −1 (strong negative correlation) to +1 (strong positive correlation), as indicated by the legend.

The correlation matrix has been visualized with significance (*P* < 0.01) in Fig. 11, offering a comprehensive overview of the relationships among clinical metrics, immune cell populations, cytokines, chemokines, and specific immune markers across the JBNU-22-N01, PJ73, VR2332, and negative control groups. In clinical parameters, serum viremia and lung viral load showed a strong positive correlation with nasal viral shedding, indicating that higher systemic viral loads are linked to increased transmission potential via respiratory routes. Average daily weight gain (ADWG) was inversely correlated with serum viremia and lung viral loads, underscoring the impact of high viral loads on growth performance. Additionally, lung cytokines (IFN-α, IFN-γ, IL-1β, and IL-10), mRNA expression levels in BAL cells (*CCL2*, *CCL8*, *CXCL10*, *CXCR5,* and *TIM3*), and notably, the serum Kyn/Trp ratio strongly correlated with ADWG, highlighting their potential as markers of virulence (Fig. 11).

The matrix reveals substantial correlations among lung viral load, alteration of immune cell populations, and associated production of cytokine/chemokine. Pre-existing alveolar macrophages (live PAM or AMɸ) showed a strong negative correlation with lung viral load, whereas monocyte-derived cells (moMɸ, cDC1, and cDC2), T cells, and NK cells were positively correlated, indicating virus-induced destruction of PAMs and host-driven recruitment of immune cells into the lung as a response to infection. Positive correlations among immune cells, chemokines (*CCL2*, *CCL5,* and *CCL8*), and chemokine receptors (CCR4, CCR5, and CXCR5) suggest chemokine-mediated cell recruitment (Fig. 11).

Immune checkpoint molecules, such as *PD1*, *PDL1*, *CTLA4*, *LAG3*, and *IDO1*, were positively correlated with fluctuations in immune cell populations and with pro-inflammatory cytokines (e.g., IFN-γ, IL-1β) and regulatory cytokine IL-10. This suggests that the upregulation of immune checkpoints may act as a regulatory mechanism to balance heightened inflammation and prevent excessive immune-mediated damage. Notably, *IDO1* expression showed a strong positive correlation with the serum Kyn/Trp ratio, affirming its role in activating the kynurenine pathway. Elevated *IDO1* was also significantly correlated with cytokines and immune checkpoint molecules potentially involved in its regulation, including IFN-γ, IL-1β, IL-6, IL-10, *PD1*, *PDL1*, *LAG3,* and *CTLA4* (Fig. S2). This supports the hypothesis that PRRSV may leverage the kynurenine pathway to induce immune tolerance or modulate inflammatory responses. Additionally, *SPP1* and *TREM2*, markers of potential tissue remodeling (48–50), were associated with cytokines and immune checkpoints, suggesting that chronic immune activation in PRRSV infection may contribute to a pro-fibrotic environment in the lung (Fig. 11).

## DISCUSSION

In the past four decades, the global spread of PRRSV-2 has led to the emergence of genetically diverse lineages with varying degrees of pathogenicity, posing a significant threat to the swine industry worldwide (9). Among these lineages, the NADC34-like viruses (L1A) have drawn particular attention due to their clinical impacts, such as reproductive losses in sows and high mortality rates in newborn piglets (14, 15), with field cases reporting abortion rates of 10%–30% and piglet mortality of 10%–40% in China (11). However, the pathogenicity of NADC34-like PRRSV in weaned piglets remains unclear, with experimental infections showing variable outcomes ranging from mild (56–59) to severe (15, 60) (Table 1).

**TABLE 1:** Animal studies investigated pathogenicity and immune responses of NADC34-like PRRSV in the weaned piglet model

Strain	Country	Isolated year	Dose (TCID_50_)	Pig age	dpi	Pathogenicity	Immune response
IA/NADC34/2014	US (15)	2014	5 × 10^4^	3 weeks old	14	Virulent	Not tested
PRRSV-ZDXYL-China-2018–1	China (57)	2018	4 × 10^3^	3 weeks old	28	Moderate	Significantly higher level of serum IFN-γ, IL-2, and IL-10 compared to low virulent strain LQ
HLJDZD32-1901	China (56)	2019	4 × 10^4^	5 weeks old	14	Mild	Not tested
JS2021NADC34	China (60)	2021	3 × 10^6^	8 weeks old	14	High	Not tested
LNSY-GY	China (58)	2022	4 × 10^5^	8 weeks old	28	Mild	Not tested
LNTZJ1341-2012	China (59)	2020	4 × 10^5^	8 weeks old	21	Mild	Not tested
JBNU-22-N01	Korea	2022	2 × 10^3^	4 weeks old	14	Moderate	Highly immunomodulatory

Precise criteria for defining PRRSV “virulence” remain unsettled, but several key indicators are often referenced (9). These include high mortality rates (>20%), severe clinical symptoms such as hyperthermia, anorexia, and reduced average daily weight gain (ADWG), and the presence of extensive interstitial pneumonia with associated inflammatory cytokine responses. Additional markers include high viral loads and a large presence of antigen-positive cells in lung tissue. For PRRSV strains to be classified as virulent, it is generally accepted that they should meet most of these criteria. According to this framework, the JBNU-22-N01 strain does not meet the criteria for "virulence" but instead exhibits moderate pathogenicity in weaned piglets, similar to previously studied PJ73 and VR2332 strains (35), as most of the indicators are at similar levels including mortality (0%), ADWG, viral loads, and the levels of cytokines, at 14 dpi, in which most of PRRSV shows its highest pathogenicity.

However, when examining the infection course, JBNU-22-N01 demonstrated a notably faster replication rate compared with other strains, consistent with previous *in vitro* findings (16). This accelerated replication was marked by elevated non-neutralizing antibody levels in serum (61) (Fig. 2) and increased systemic levels of IFN-α, a type I interferon known to rise in parallel with viral load during early PRRSV infection (9, 62) (Fig. 8A). Additionally, JBNU-22-N01 infection led to more severe lung lesions at an earlier stage (seven dpi), characterized by high viral load and interstitial pneumonia, which corresponded with increased PRRSV antigen detection (Fig. 4; Table S4). This replication profile suggests that JBNU-22-N01 follows a distinct course of disease progression, marked by rapid viral expansion and an intense early immune response.

In JBNU-22-N01-infected bronchoalveolar lavage (BAL) cells, the chemokines CCL2 (MCP-1), CCL5 (RANTES), CCL8 (MCP-2), and CXCL10 (IP10) were upregulated at 7 dpi, consistent with previous transcriptomic studies showing their elevation in PRRSV-infected BAL cells (Fig. 9A) (20, 28). These chemokines likely facilitate faster lymphocyte and monocyte-derived cell infiltration into the lungs, reflected by earlier upregulation of chemokine receptors CCR4, CCR5, and CXCR5 (Fig. 9A), than seen with PJ73 and VR2332, helping counter viral infection and restore lung homeostasis (21, 24, 28, 42, 43) (Fig. 5). As a proinflammatory chemokine, CXCL10 can either be protective or pathogenic in viral infections, often correlating with disease severity in respiratory syndromes such as SARS and MERS (42). In this study, CXCL10 mRNA expression in BAL cells was strongly correlated with increased proinflammatory cytokines IFN-α, IFN-γ, IL-1β, and TNF-α, all known to drive interstitial pneumonia progression (9, 27, 62), and negatively correlated with ADWG, suggesting its potential role as a virulence marker in PRRSV-2 infection (Fig. 11) (20).

In highly virulent PRRSV-1 Lena infections, elevated proinflammatory cytokines have been strongly linked to severe lung damage (33). Likewise, JBNU-22-N01 infection showed significantly increased levels of lung IFN-α, IFN-γ, IL-1β, and IL-12p40 at 7 dpi, indicating a faster disease progression and host response than PJ73 or VR2332 (Fig. 8B), which is likely to be correlated with faster virus replication (Fig. 11). Interestingly, unlike previous studies that reported upregulation of IL-6 during highly virulent PRRSV infections such as HP-PRRSV (9, 27, 62), IL-6 levels in this study gradually decreased, particularly in JBNU-22-N01 infection (Fig. 8C). Similarly, HMGB1 levels also decreased, showing a strong correlation with the reduction in IL-6 (Fig. 8D). These findings contrast with earlier reports that observed HMGB1 upregulation in HP-PRRSV-infected lungs (41). Notably, a previous *in vitro* transcriptomic investigation comparing Chinese NADC34-like PRRSV strain YC-2020 with HP-PRRSV strain JXA1 revealed that NADC34-like PRRSV exhibited significantly lower expression of inflammatory-associated genes than HP-PRRSV. However, the YC-2020 strain showed higher expression of genes related to type I interferon, IL-1, CCL4, TNF, and NF-κB, suggesting a distinct gene expression profile (63). Based on these findings, the lung cytokine profiles of IL-6 and HMGB1 may serve as potential markers of virulence to differentiate between lethal (e.g., HP-PRRSV) and non-lethal (e.g., VR2332, JBNU-22-N01) PRRSV infections. Although the underlying mechanism driving the decline in HMGB1 and IL-6 remains unclear, it is possible that PRRSV-induced PAM depletion (Fig. 5) and M2-like polarization, evidenced by increased expression of SPP1 and TREM2 (Fig. 9D), contributed to this decline. The shift toward M2-like macrophage polarization, which could be associated with the resolution phase of PRRSV-induced inflammation and reduced proinflammatory cytokine production (Fig. 8), may have led to decreased secretion of IL-6 and HMGB1 in the infected lungs. However, at the same time, despite the well-characterized proinflammatory effects of HMGB1, including the stimulation of IL-1β and IL-8 (41), these cytokines did not exhibit a corresponding decrease in PRRSV-infected lungs, particularly in the JBNU-22-N01 group, where HMGB1 levels were markedly reduced. This apparent inconsistency suggests a more complex regulatory network *in vivo*, in which the production of specific cytokines may be influenced by multiple variables beyond HMGB1 alone, such as viral interference with PRR signaling, cytokine-specific regulatory loops, or the differential cellular sources of cytokines (64). It is also possible that the redox status or subcellular localization of HMGB1 altered its functional activity, as previously reported (40). Further studies are warranted to clarify whether this decline stems from active immune regulation or is simply a downstream effect of PAM depletion.

Interferon-stimulated genes (ISGs), induced by IFNs, play a crucial role in IFN signaling (65). Their protein products, such as Mx protein and NOS, are known to restrict virus replication (66, 67). Previous studies have identified ISGs such as ISG15, ISG12(A), MX1, and MX2 as inhibitors of PRRSV replication in MARC-145 cells (45–47, 68). However, PRRSV NSPs can antagonize ISG15, counteracting its antiviral effects (44). In this study, despite significant production of IFNs in the lung during the infection, ISG15, ISG12(A), MX1, and MX2 genes were downregulated at 14 dpi (Fig. 9B), suggesting that PRRSV employs strategies to inhibit ISGs and facilitate *in vivo* replication. Interestingly, ISG12(A) was upregulated at 7 dpi in all infection groups. ISG12(A), induced by IFN-α and IFN-γ (69), has been identified as an antiviral ISG capable of significantly restricting infections such as hepatitis C virus (HCV) (70), and Newcastle disease virus (NDV) (71). Conversely, ISG12(A) also functions as a pro-apoptotic protein involved in the caspase-dependent pathway (72), inducing cytochrome c release, Bax activation, and mitochondrial membrane destabilization to enhance apoptosis (73). Porcine ISG12(A), localized in mitochondria, has been shown to arrest MARC-145 cells in the G2/M phase (46). Thus, it has been suggested that PRRSV-induced ISG12(A) may promote apoptosis, eliminating host cells to prevent viral replication and dissemination (46). In this context, the upregulation of ISG12(A) at 7 dpi may drive the subsequent decreased frequency of pre-existing bystander PAMs at the peak lung damage timepoint (14 dpi) (Fig. 5), supported by the fact that PRRSV directly infects less than 2% of PAMs (19–21, 23).

Along with the cytokine and chemokine cascade, JBNU-22-N01 infection led to a more rapid decline in the frequency of PAMs, the virus’s primary target cells, resembling the pattern observed in highly virulent PRRSV-1 Lena infection compared with less virulent PRRSV-1 3249 strain (21, 28) (Fig. 5). The decreased frequency of PAMs was associated with a decrease in MHCII^+^ cells (Fig. 5), consistent with previous reports of PRRSV-mediated impairment of antigen presentation through MHCII downregulation (24). In response to PAM loss, MHCII^+^ monocyte-derived macrophages (moMΦ) and dendritic cells (moDC, cDC1, cDC2) increased in correlation with lung viral load (24, 74) and chemokines (Fig. 5 and 11). Additionally, increased mRNA expression of TREM2 and SPP1, potential markers for M2 macrophage polarization (49, 50) and tissue remodeling (48), respectively, further indicated changes in myeloid cell profiles in the lung during PRRSV-2 infection (Fig. 9D).

Simultaneously, a notable influx of T lymphocytes and NK cells was observed in BAL, consistent with the previous study (24), in correlation with chemokines and chemokine receptors (Fig. 5, 6, and 11). During the peak of immune cell fluctuations, the proportion of CD8^+^ T cells (9-15%) was higher than that of CD4^+^ T cells (3%–5%) (Fig. 5 and 6). However, effector T cells (Th1, Th17, and CTLs), assessed by intracellular cytokine staining (ICS) after antigen stimulation, constituted only a minor proportion (< 1%) (Fig. 6), which was consistent with the previous report (75), suggesting a potentially dysregulated T cell response. Although JBNU-22-N01 infection triggered early T cell influx at 7 dpi and increased populations of effector T cells and Tregs at 14 dpi, the proportion of effector T cells remained low (< 2%) relative to the total infiltrating CD4^+^ and CD8^+^ T cells (Fig. 5 and 6). The upregulation of FoxP3^+^ Tregs and IL-10 in the lung tissue is known to delay the Th1 response by suppressing antigen-presenting cell (APC)-mediated CD4^+^ T cell proliferation (25, 27) (Fig. 6 and 8B). However, the precise mechanism for suppressed naïve CD8^+^ T cell activation could not be fully explained by those indicators, necessitating further exploration into immune checkpoint molecules as potential regulators.

Growing evidence suggests that PRRSV infection leads to the upregulation of immune checkpoint molecules, such as PD1, PDL1, CTLA4, and IDO1, in the lungs and BAL cells (20, 23, 28, 33). Notably, this phenomenon has been reported consistently across various PRRSV genotypes and lineages, including both PRRSV-1 and PRRSV-2, suggesting a conserved immunomodulatory mechanism. Furthermore, the increased expression of these molecules is not limited to the lungs but has also been observed in non-primary PRRSV replication sites, such as the thymus (32) and inguinal lymph node (34), implying that immune checkpoint regulation may contribute to systemic immunomodulation during PRRSV infection. However, a major limitation, including in the present study, is that most analyses relied on bulk RNA measurements from tissues or BAL cells, which consist of heterogeneous cell populations. As a result, it remained unclear whether PRRSV-induced upregulation of immune checkpoint molecules is truly cell-type specific. To address this question, our group recently performed single-cell transcriptomic analysis of BAL cells from PRRSV-infected pigs with varying pathogenicity (23). This analysis revealed cellular infiltration patterns as well as kinetics of Kyn/Trp ratio and IDO1 expression, highly consistent with those observed in the present study. Most importantly, it demonstrated that immune checkpoint molecule expression is specifically upregulated in infiltrating T cells at the single-cell level. In this context, elevated immune checkpoint expression, together with ICS-based detection of low cytokine-producing T cells, supports a hypothesis of dysregulated T-cell activity. Nevertheless, further studies involving T cell functional assays such as proliferation or recall response assays would be required to draw definitive conclusions about T-cell dysfunction during PRRSV infection.

T cell activation requires both TCR-MHC binding and costimulatory signals, such as T cells expressing CD28 interacting with APC-expressed CD80/CD86, to drive T cell proliferation, survival, and differentiation (76). Immune checkpoint molecules, including CTLA4, PD1, and LAG3, serve crucial roles in immune homeostasis by modulating immune responses and preventing excessive tissue damage, which is often exploited by cancers and pathogens to escape immune control (29). For instance, T cells expressing CTLA4 bind CD80/86 with greater affinity than CD28, leading to T cell anergy by functioning at the priming phase of T cell activation (76, 77). CTLA4 is also constitutively expressed on Tregs and is important for their suppressive function (76). PD1, more broadly expressed on activated T cells, B cells, and myeloid cells, is a member of the B7/CD28 family of costimulatory receptors, and it regulates T cell activation through binding to its ligands, PDL1 and PDL2 (76, 77). When a T cell experiences coincident TCR and PD1/PDL1 binding during the effector phase, predominantly in peripheral tissue, PD1-generated signals prevent phosphorylation of key TCR signaling intermediates, terminating early TCR signaling and reducing activation of T cells (76, 77). LAG3 structurally resembles the CD4 coreceptor and binds to MHCII with a higher affinity than CD4 and has been found briefly upregulated in activated CD4^+^ and CD8^+^ T cells, exhausted CD8^+^ T cells, Tregs, type 1 regulatory T cells, and NK cells (33, 78). Under transcriptomic investigation, PDL1 and IDO1 were only upregulated in PRRSV-infected PAMs compared with non-infected PAMs, suggesting that active viral replication is necessary for the induction of those molecules (20). In this study, all PRRSV-2 infections upregulated checkpoint molecules including PD1, PDL1, CTLA4, LAG3, and IDO1 in infected BAL cells at peak lung damage time point (14 dpi), resembling responses seen in virulent PRRSV-1 strains (33), indicating that checkpoint-mediated T cell regulation is the common immunosuppressive pathway shared by PRRSV infections (Fig. 9C). Earlier and higher upregulation of these molecules in JBNU-22-N01 infection might be induced by higher proinflammatory cytokine cascade and earlier infiltration of immune cells (Fig. 5 and 8), which was similar to highly virulent PRRSV-1 Lena infection (28, 33), suggesting that upregulation of immune checkpoints is necessary to suppress excessive inflammatory responses by shifting from a proinflammatory to an immunosuppressive lung environment, thus protecting tissues but adversely impairing activation of infiltrated naïve T cells in a synergistic manner.

IDO1, an intracellular immune checkpoint expressed in APCs, catalyzes Trp degradation via the kynurenine pathway, resulting in Trp depletion and Kyn metabolite accumulation. This dual effect contributes to immune regulation by impairing effector immune cells and promoting immunosuppressive phenotypes (30, 31, 53). Trp depletion activates the GCN2 and mTOR pathways (79), suppressing CD8^+^ T cell proliferation and promoting FoxP3^+^ Treg differentiation through mTORC1 inhibition and GCN2 signaling (52, 53, 79). Additionally, Kyn activates AhR, which drives Treg differentiation, PD1 upregulation, and a tolerogenic phenotype in macrophages (80). Clinically, IDO1-driven Trp breakdown, reflected by altered Kyn/Trp ratios, correlates with immune suppression and poor prognosis in tumors and viral infections (30, 54, 55). In this study, high Kyn/Trp ratios were associated with reduced ADWG, suggesting a clinical role of IDO1 and Trp metabolism in PRRSV infection (Fig. 11). In pigs, Trp serves as a serotonin precursor and is crucial for feed intake regulation. Trp deficiency, therefore, reduces appetite and feed intake, adversely affecting growth (81, 82). Despite limited studies on circulating Trp and Kyn in PRRSV-infected pigs, dietary Trp supplementation has been shown to mitigate feed intake reduction following PRRSV vaccination (83). Although our study showed increased IDO1 mRNA and protein expression in BAL cells, particularly at 14 dpi, the corresponding serum Kyn/Trp ratios did not consistently correlate with IDO1 protein levels across groups (Fig. 10). This discrepancy highlights the complex regulation of tryptophan metabolism *in vivo*. Serum Kyn/Trp ratios may reflect cumulative IDO1 activity from multiple tissues, not solely the lung, and IDO1 enzymatic function may also be modulated post-transcriptionally by inflammatory cues such as IFN-γ or TNF-α. Nevertheless, the observed trends suggest that IDO1-mediated tryptophan catabolism could play an immunomodulatory role in the lung microenvironment and systemically, potentially affecting effector T cells, monocytes, and NK cells. This is further supported by the increased proportion of FoxP3^+^ cells in BAL (Fig. 6), as well as the delayed systemic T cell response observed in PBMCs (Fig. 7). Although these findings do not establish direct causality, they are consistent with previous reports on IDO1-driven immune modulation in viral infections and warrant further investigation through enzyme activity or metabolomics assays.

IDO1 expression is primarily induced by IFN-γ via NF-κB and STAT-1 pathways (84, 85). Proinflammatory cytokines, such as IL-1β and TNF-α, further amplify this effect by enhancing IFN-γ receptor (IFN-γR) expression on APCs via NF-κB-mediated mechanisms (86). Additionally, PRRSV infection increases TLR3 and TLR7 expression, which activates NF-κB (87, 88), potentially further driving IDO1 expression, as observed in SARS-CoV-2 infections (84). Beyond cytokine regulation, CTLA4-CD80/86 interactions stimulate IDO1 expression, whereas CD28-CD80/86 interactions promote IL-6-dependent IDO1 degradation, illustrating a balance in its regulation (30, 89). Additionally, AhR activated by Kyn could sustain IDO1 expression via an AhR-IL-6-STAT3 feedback loop, as seen in tumors (90). In this study, IFN-γ, IL-1β, and TNF-α levels were positively correlated with IDO1 mRNA expression in BAL cells under PRRSV infection (Fig. 11; Fig. S2). Notably, IL-6 levels negatively correlated with CTLA4 and IDO1 expression, particularly in JBNU-22-N01 infection (Fig. 8B and 11). These findings suggest that PRRSV-induced IDO1 expression is driven by proinflammatory cytokines and CTLA4-CD80/86 interactions, potentially extending IDO1 stability by modulating IL-6 pathways. It is consistent with prior observations in highly virulent PRRSV-1 Lena infections, where CTLA4 expression was upregulated to a greater extent than CD28 expression in PRRSV-infected lung, indicating an imbalance favoring IDO1 induction (33). Further *in vitro* studies focusing on IDO1 inducers, AhR activation, and metabolic regulation are needed in the future to elucidate the underlying mechanisms of PRRSV-induced IDO1 production and Trp catabolism.

In summary, based on the kinetics of viral replication and the host immune responses (Fig. 11), we propose that one of the main mechanisms of immunosuppression during the early phase of PRRSV-2 infection, following proinflammatory cytokine/chemokine cascade, is the immune cell regulation mediated by cell-to-cell communications between T cells and APCs through immune checkpoints and IDO1-linked metabolites, resulting in or from IDO1 production in a synergistic manner. Regarding viral phenotype, we classify the NADC34-like PRRSV as not only moderately pathogenic but also highly immunomodulatory, with a faster replication rate that generates a distinct immunological profile characterized by intensified immune activation, elevated proinflammatory cytokines, immune checkpoint expression, and increased kynurenine pathway activity marked by IDO1 upregulation. This strong immunomodulatory capacity may explain the variable clinical outcomes observed in previous animal studies (Table 1), with differences in virulence potentially linked to the age and immune status of infected pigs and the presence of co-infections. The virus’s rapid replication and pronounced immunomodulatory effects likely contribute to its more severe clinical impact in pregnant sows and newborn piglets, which are more vulnerable due to pregnancy and immature immune systems.

## Data Availability

The data sets used and/or analyzed during the current study are available from the corresponding author on reasonable request.

## SUPPLEMENTAL MATERIAL

Supplemental material jvi.02298-24-s0001.pdf Tables S1 to S4; Fig. S1 and S2.

ASM does not own the copyrights to Supplemental Material that may be linked to, or accessed through, an article. The authors have granted ASM a non-exclusive, world-wide license to publish the Supplemental Material files. Please contact the corresponding author directly for reuse.
