# Supplementary material for: The role of immune checkpoint molecules in PRRSV-2-induced immune modulation: insights from comparative in vivo evaluation including NADC34-like PRRSV
Source: J Virol. 2025 Jun 3;99(7):e02298-24. doi: 10.1128/jvi.02298-24 (PMC12282115; doi:10.1128/jvi.02298-24)
Supplement: Supplemental material — Tables S1 to S4; Fig. S1 and S2. [file jvi.02298-24-s0001.pdf]

Supplementary Table 1. Microscopic lesion scoring system in this study

Lung lesions (Interstitial pneumonia [PRRS] + inflammation)		
**Score**	**Interstitial pneumonia (PRRS)**	**Inflammation**
0	No microscopic lesions	No microscopic lesions
1	Mild interstitial pneumonia	mild suppurative pneumonia or pleuritis under 25% lung lobe involvement
2	Moderate multifocal interstitial pneumonia	suppurative pneumonia or pleuritis with 25 to 50% lung lobe involvement
3	Moderate diffuse interstitial pneumonia	suppurative pneumonia or pleuritis with 50 to 75% lung lobe involvement
4	Severe interstitial pneumonia	suppurative pneumonia or pleuritis with over 75% lung lobe involvement

Supplementary Table 2. List of cell-staining panels and used antibodies

panel	antibody	provider	catalog number (clone number)
Live/ dead DC/ macrophage	Zombie Green live-dead fixable dye	BioLegend	423112
	Mouse anti-swine MHCII IgG2a	Kingfischer Biotech	WS0589S-100
	Mouse anti-pig CD163 IgG1	Bio-Rad	MCA2311GA (2A10/11)
	Mouse anti-pig CD172a PE	Bio-Rad	MCA6100PE (74-22-15)
	Rat anti-mouse IgG1 APC	BioLegend	406610
	Rat anti-mouse IgG2a PerCP Cy5.5	BioLegend	407112
CD3/ CD4/ CD8/ gammadelta T	Mouse anti-pig CD3 FITC	Bio-Rad	MCA5951F (PPT3)
	Mouse anti-pig CD4a PerCP Cy5.5	BD	561474 (74-12-4)
	Mouse anti-pig CD8a PE	BD	559584 (76-2-11)
	Mouse anti-swine TCR1 Delta-chain IgG1	Kingfischer Biotech	WS0621S-100
	Rat anti-mouse IgG1 APC	BioLegend	406610
Natural killer (NK) cell	Mouse anti-pig CD3 FITC	Bio-Rad	MCA5951F (PPT3)
	Mouse anti-pig CD8a PE	BD	559584 (76-2-11)
	Mouse anti-pig CD335 (NKp46) IgG1	Bio-Rad	MCA5972GA (VIM-KM1)
	Rat anti-mouse IgG1 APC	BioLegend	406610
Regulatory T (Treg) cell	Mouse anti-pig CD4a PerCP Cy5.5	BD	561474 (74-12-4)
	Mouse anti-pig CD8a PE	BD	559584 (76-2-11)
	Mouse anti-pig CD25 IgG1	Bio-Rad	MCA1736GA (K231.3B2)
	Rat anti-FoxP3 FITC	eBioscience	11-5773-82 (FJK-16s)
Cytotoxic T (CTL) cell	Mouse anti-pig CD3 FITC	Bio-Rad	MCA5951F (PPT3)
	Mouse anti-pig CD8a PE	BD	559584 (76-2-11)
	Mouse anti-pig IFN-γ PerCP Cy5.5	BD	561481 (P2G10)
T helper 1, 17 (Th1, Th17) cell	Mouse anti-pig CD4 PE	BD	559586 (74-12-4)
	Mouse anti-pig CD8 FITC	BD	551303 (76-2-11)
	Mouse anti-pig IFN-γ PerCP Cy5.5	BD	561481 (P2G10)
	Mouse anti-IL17 APC	eBioscience	17-7179-42 (eBio64DEC17)

Supplementary Table 3. Information of the primers used for mRNA quantification in BAL cells

Target gene	GenBank accession no.	Primer	Sequence (5'-3')	Amplicon size (bp)	Remark	Sequence reference
HPRT1	NM_001032376.2	HPRT1F	GGACTTGAATCATGTTTGTG	91	Housekeeping gene	Nygard et al., 2007
		HPRT1R	CAGATGTTTCCAAACTCAAC			
CCL2	NM_214214.1	CCL2F	GCGGCTGATGAGCTACAGAAG	75	Chemokine	Jabs et al., 2007
		CCL2R	CCCGCGATGGTCTTGAAG			
CCL5	NM_001129946.1	CCL5F	ACACCACACCCTGCTGTTTT	150	Chemokine	This study (NCBI primer blast)
		CCL5R	TCTTCTCTGGGTTGGCACAC			
CCL8	NM_001164515.1	CCL8F	GGTGCTTGCTCAGCCAGATT	118	Chemokine	This study (NCBI primer blast)
		CCL8R	ACACTGGCTGTTGGTGATTCT			
CXCL10	NM_001008691	CXCL10F	TGCAGCACCATGAACCAAAG	109	Chemokine	This study (NCBI primer blast)
		CXCL10R	TGATGCAGGTACAGCGAACA			
CCR4	XM_003361792.1	CCR4F	TTCACTTCCAGGGCTTTTGTTC	95	Chemokine receptor	This study (NCBI primer blast)
		CCR4R	CCTTCCATCTTGTGGAGTTGAAA			
CCR5	NM_001001618.1	CCR5F	CCAGGCAGAGGCTCCAGAT	78	Chemokine receptor	Jabs et al., 2007
		CCR5R	CACAAGCCGACAGAGATTTCC			
CXCR5	XM_003129915.1	CXCR5F	CCTCCCGCTTTCTCTACCAC	75	Chemokine receptor	This study (NCBI primer blast)
		CXCR5R	CCACCCCTACGTAACACCAG			
ISG15	NM_001128469.3	ISG15F	AGCAACGCCTATGAGGTCTG	107	Interferon-stimulated gene	Liu et al., 2020
		ISG15R	CCCTCGAAAGTCAGCCAGAA			
ISG12(A)	NM_001198921.1	ISG12AF	CCAAGATACTGGCGACAGGG	114	Interferon-stimulated gene	This study (NCBI primer blast)
		ISG12AR	CGGTTAGGGCAGCCTTGAAT			
MX1	NM_214061.2	MX1F	AGTTACCGGGACAGCGAGAT	103	Interferon-stimulated gene	This study (NCBI primer blast)
		MX1R	GACTGATTCCCACGCCTTCC			
MX2	NM_001097416.1	MX2F	TGGAGCGGGAAATACGCAA	116	Interferon-stimulated gene	This study (NCBI primer blast)
		MX2R	GATGAGGGTCAGGTCTGGAAC			
PD1	NM_001204379.1	PD1F	AGCCCAAGCACTTCATCCTC	147	T cell exhaustion marker	Ruedas-Torres, I. et al. 2021
		PD1R	TGTGGAAGTCTCGTCCGTTG			
PDL1	XM_005660125	PDL1F	GTGGAAAAATGTGGCAGCCG	140	T cell exhaustion marker	
		PDL1R	TGCTTAGCCCTGACGAACTC			
PDL2	NM_001025220.1	PDL2F	GGGCAGTATCGCTGCTTCAT	107	T cell exhaustion marker	
		PDL2R	ACCTTGAGGTTGCGAGTCTT			
CTLA4	NM_214149.1	CTLA4F	TCTTCATCCCTGTCTTCTCCAAA	103	T cell exhaustion marker	
		CTLA4R	GCAGACCCATACTCACACACAAA			
CD200R1	NM_001243546.1	CD200R1F	TGTTCCAAGTTACTAATCAGGCTGAA	84	T cell exhaustion marker	
		CD200R1R	AGCCCATTAGCAACATGATACTCTTT			
LAG3	NM_001105306.1	LAG3F	CTCCTCCTGCTCCTTTTGGTT	132	T cell exhaustion marker	
		LAG3R	CAGCTCCCCAGTCTTGCTCT			
TIM3	XM_003134109.4	TIM3F	AGGGCAGGACACAGTCAAAG	103	T cell exhaustion marker	
		TIM3R	AGGGCAGGACACAGTCAAAG			
IDO1	NM_001246240.1	IDO1F	GGCACTTGATTGGTGGTCTC	132	T cell exhaustion marker	
		IDO1R	GCAATCCAAGCATCGTAAGG			
SPP1	NM_214023	SPP1F	TAATTCTGGCAGCTCGGAGG	108	potential lung disease progression marker	This study (NCBI primer blast)
		SPP1R	TGTGGCGCTAGGAAAGTCTG			
TREM2	NM_001256777.1	TREM2F	CACACTCACCATTACGCTGC	125	potential M2 macrophage marker	This study (NCBI primer blast)
		TREM2R	GACCTTCTTGAGGGTGTCGG			

Supplementary Table 4. Full description of histopathological findings

Group	dpi	Pig #	Histopathology					Interstitial pneumonia score	Inflammation score	Total microscopic score	IHC score
			Perivascular cuffing	Necrotic cells in the alveoli	Pleurisy	Pneumonia					
						Broncho	Interstitial				
Negative control	7	1	-	-	-	-	-	0	0	0	0
	7	2	++	-	-	-	+	1	0	1	0
	7	3	-	-	-	-	-	0	0	0	0
VR2332	7	7	+	+	-	-	++	2	0	2	2
	7	8	-	-	-	-	-	0	0	0	1
	7	9	+	-	-	-	+	1	0	1	1
JB15-N-PJ73-GN (PJ73)	7	13	+	+	-	-	++	2	0	2	2
	7	14	+	-	-	-	-	0	0	0	0
	7	15	-	-	-	-	+	1	0	1	0
JBNU-22-N01	7	19	+	++	-	-	++	2	0	2	3
	7	20	++	++	-	-	+++	3	0	3	2
	7	21	+	+++	-	-	+++	3	0	3	4
Negative control	14	4	-	-	-	-	+	1	0	1	0
	14	5	+	-	-	-	+	1	0	1	0
	14	6	+	-	-	-	-	0	0	0	0
VR2332	14	10	++	+	+	-	+++	3	1	4	3
	14	11	-	+	-	-	++	2	0	2	3
	14	12	++	++	-	-	+++	3	0	3	1
JB15-N-PJ73-GN (PJ73)	14	16	++	+	-	-	+++	3	0	3	3
	14	17	+	+	+	-	+++	3	1	4	2
	14	18	++	+++	-	-	++++	4	0	4	3
JBNU-22-N01	14	22	++	-	-	-	+++	3	0	3	3
	14	23	++	-	+	-	+++	3	1	4	2
	14	24	++	++	-	-	++++	4	0	4	4

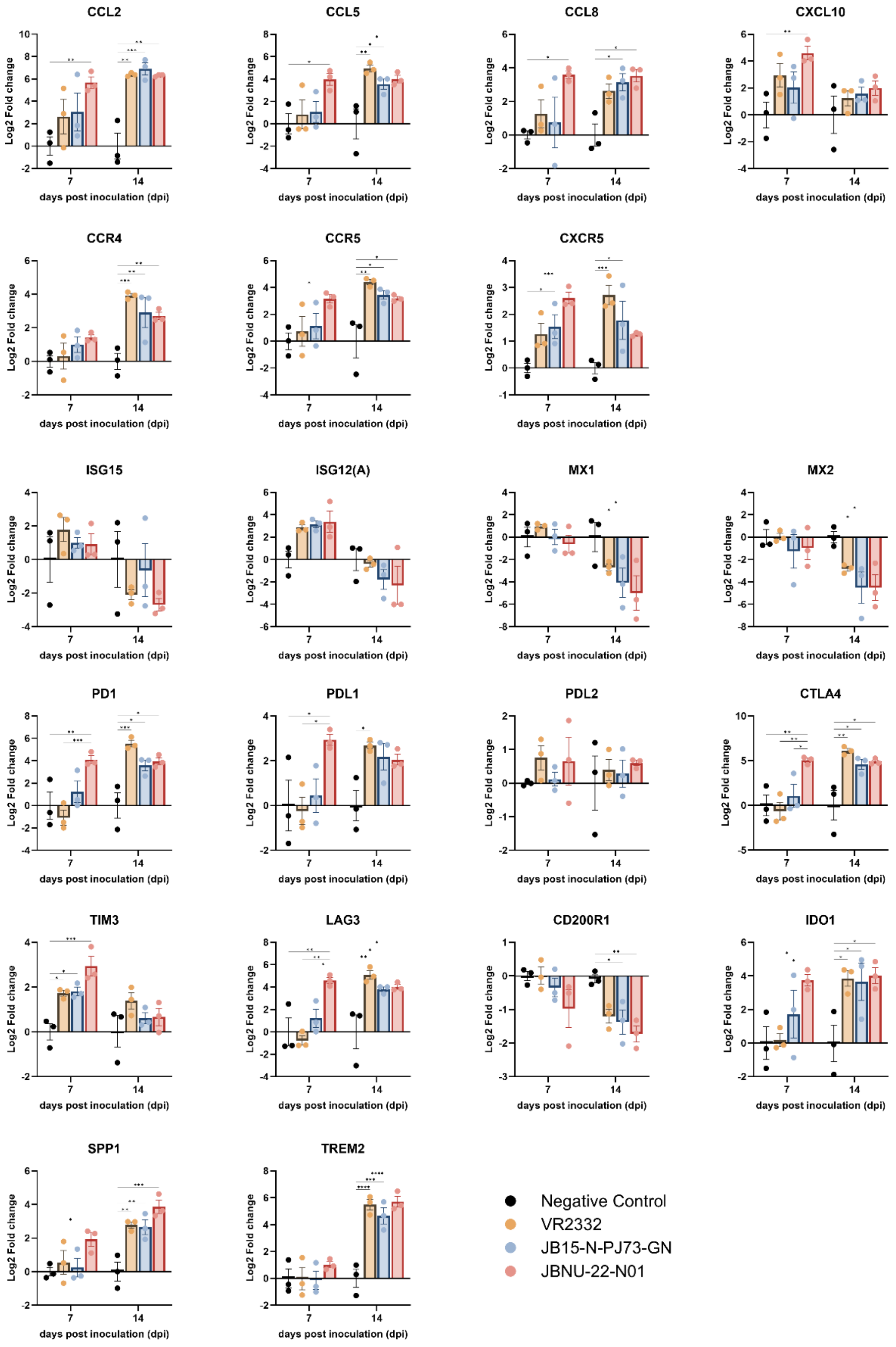

Supplementary Figure 1. Bar plots showing mRNA expressions in BAL cells.

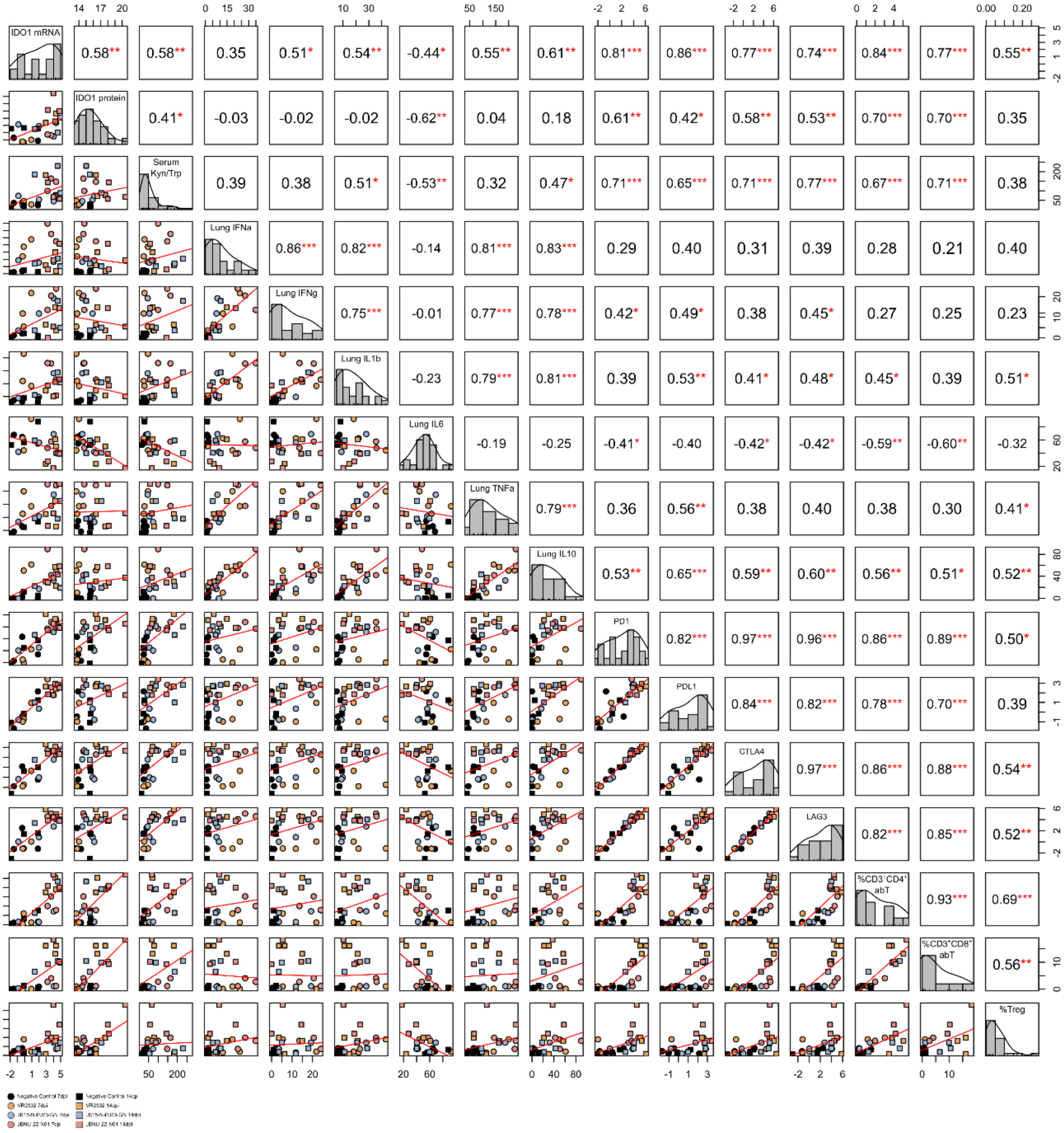

Supplementary Figure 2. Spearman correlation matrix with regulators potentially linked to IDO1 upregulation.
